# Hyperglycemia aggravates vitiligo through succinate/SUCNR1-mediated T cell activation

**DOI:** 10.1172/JCI200316

**Published:** 2026-06-15

**Authors:** Pan Kang, Yuqian Chang, Tingting Wang, Xiuli Yi, Yinghan Wang, Pengran Du, Jiaxi Chen, Baizhang Li, Shuli Li, Zhongjun Shao, Jianru Chen, Chunying Li

**Affiliations:** 1 Department of Dermatology, Xijing Hospital, and; 2 Department of Epidemiology, School of Public Health, Fourth Military Medical University, Xi’an, Shaanxi, China.; 3 National Key Laboratory of Immunity and Inflammation, Institute of Immunology, Naval Medical University, Shanghai, China.

**Keywords:** Autoimmunity, Dermatology, Immunology, Glucose metabolism, Skin, T cells

## Abstract

Vitiligo is an autoimmune skin disease characterized by depigmentation, mainly due to CD8^+^ T cell–mediated destruction of melanocytes. Hyperglycemia exacerbates autoimmune responses and is associated with vitiligo; however, the underlying immunometabolic mechanisms are poorly understood. Here, we demonstrated the correlation between hyperglycemia and vitiligo in a case-control study and demonstrated that hyperglycemia aggravated vitiligo based on a mouse model. Targeted metabolomics identified succinate as the potential metabolite mediating hyperglycemia-aggravated vitiligo. Mechanistically, succinate promotes the activation of CD8^+^ T cells through succinate receptor 1 (SUCNR1) and promotes keratinocytes to secrete CXCL9 and CXCL10 by enhancing the stability and nuclear translocation of hypoxia-inducible factor-1α, facilitating the skin-homing of CD8^+^ T cells. Thus, hyperglycemia aggravates vitiligo through succinate/SUCNR1 axis–regulated CD8^+^ T cell hyperactivation. Our study provides insights into the long-observed yet previously unclear mechanism by which hyperglycemia accelerates vitiligo progression and highlights SUCNR1 as a potential therapeutic target.

## Abstract 2

Hyperglycemia aggravates vitiligo through succinate, a metabolite that facilitates CD8^+^ T cell hyperactivation and skin homing, explaining the documented clinical association between hyperglycemia and autoimmune skin disease.

## Graphical abstract

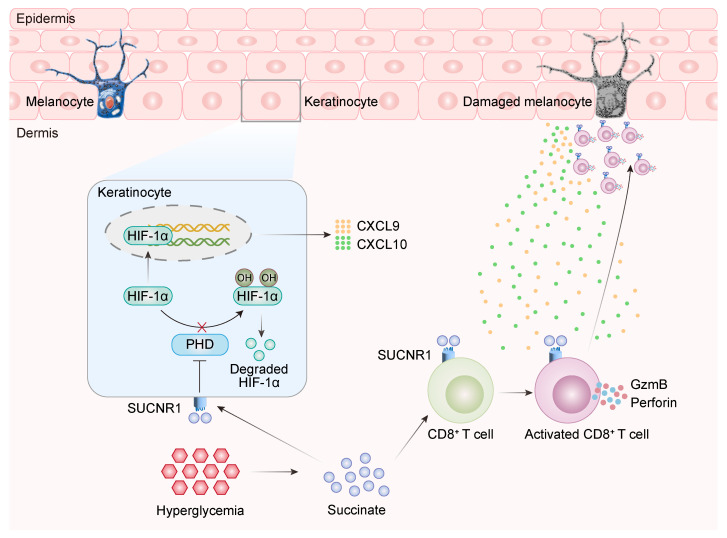

## Introduction

Vitiligo is a chronic inflammatory skin disease characterized by cutaneous depigmentation resulting from the destruction of melanocytes (1, 2). Hyperactivated CD8^+^ T cells serve as the primary effector cells of melanocyte destruction in the pathogenesis of vitiligo (1, 3). However, the triggers and mechanisms of CD8^+^ T cell hyperactivation have not been fully elucidated.

Increasing evidence supports the role of metabolic homeostasis as the regulator of immune response (4). A series of epidemiological studies have shown that vitiligo is often accompanied by disordered glucose metabolism (5–9). Our previous meta-analysis also demonstrated that hyperglycemia is associated with vitiligo and was in agreement with others (10, 11). Additionally, hyperglycemia reportedly aggravates various autoimmune diseases like colitis and experimental autoimmune encephalomyelitis (12, 13). However, whether hyperglycemia drives vitiligo progression and the underlying mechanisms remain to be fully elucidated.

Succinate, an intermediate metabolite of the glucose-driven tricarboxylic acid (TCA) cycle, plays a crucial role in adenosine triphosphate generation in mitochondria. Extracellular succinate accumulating in circulation or local tissues functions as a pro-inflammatory mediator in conditions of autoimmune or inflammatory diseases, such as rheumatoid arthritis (14, 15), inflammatory bowel disease (16, 17), and aortic aneurysm and dissection (18). Of note, succinate levels are also found to be increased in patients with diabetes (19) and mouse models (20), and glucose enhances succinate secretion in a concentration-dependent manner in vitro (19). In addition, plasma succinate levels were higher in patients with vitiligo according to nontargeted metabolomic assays (21). Therefore, succinate may serve as a potential pathogenic metabolite in vitiligo. The relationship between succinate and hyperglycemia-modified vitiligo, as well as its underlying pathogenesis, remains unclear and merits further analysis.

Succinate receptor 1 (SUCNR1), also known as G protein–coupled receptor 91 (GPR91), serves as a specific membrane receptor for succinate (22–24). Generally, SUCNR1 is assumed to be functionally inactive in healthy tissues and has been mainly investigated in inflammatory conditions (25, 26), particularly in those associated with chronic hypersuccinemia (27–29). Extracellular succinate is found to exacerbate autoimmune diseases like endometritis (30), rheumatoid arthritis (15), and inflammatory bowel disease (16) by activating SUCNR1 to regulate the pro-inflammatory response of macrophages (15, 30, 31) and the function of regulatory T cells (Tregs) (16). Based on these findings, we hypothesized that succinate/SUCNR1 axis–mediated immune disturbance is involved in hyperglycemia-regulated progression of vitiligo. However, the exact mechanisms of this process warrant further clarification.

To test this hypothesis, we initially examined the association between hyperglycemia and vitiligo in clinical samples and vitiligo mouse models in vivo. Subsequently, we screened metabolites that mediate the regulation of hyperglycemia in vitiligo. Finally, we focused on succinate and clarified the mechanisms underlying the hyperglycemia-regulated succinate/SUCNR1 axis in the pathology of vitiligo.

## Results

### Hyperglycemia promotes the progression of vitiligo by enhancing CD8^+^ T cell immune response.

**Figure 1:**
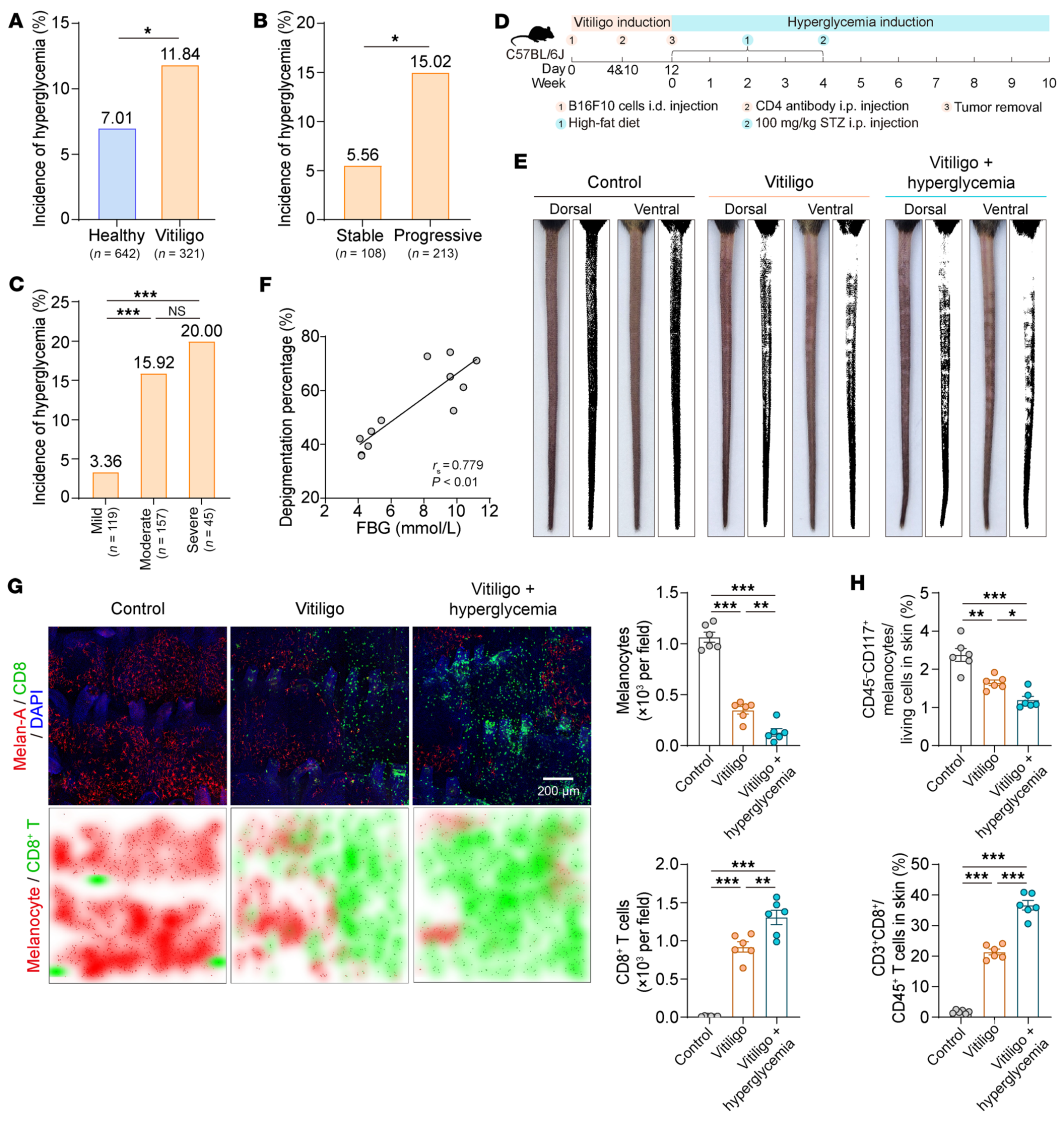
Hyperglycemia aggravates vitiligo. (**A**–**C**) Incidence of hyperglycemia in vitiligo patients and healthy controls (**A**), vitiligo patients with different activity (**B**), and vitiligo patients with different severity (**C**). (**D**) Schematic presentation of the vitiligo and hyperglycemia mouse model establishment. Thus, 3 groups of mice (Control, Vitiligo, and Vitiligo + Hyperglycemia) were studied here. (**E**) Representative tail images of mice in each group at week 10. (**F**) The correlation between tail depigmentation percentage and FBG levels of vitiligo-induced mice at week 10 (*n* = 12). (**G**) Representative whole-mount immunofluorescence images and quantification of melanocytes (shown in red) and CD8^+^ T cells (green) in tail epidermis of mice in each group (*n* = 6). (**H**) Quantification of CD45^–^CD117^+^ melanocytes and CD3^+^CD8^+^ T cells in tail epidermis of mice in each group (*n* = 6). Data are presented as frequency (**A**–**C**) or mean ± SEM (**G** and **H**) and analyzed by χ^2^ test (**A**–**C**) or 1-way ANOVA (**G** and **H**). *P < 0.05; **P < 0.01; ***P < 0.001. FBG, fasting blood glucose.

We initially conducted a hospital-based case-control study to verify the association between hyperglycemia and vitiligo. Participants’ demographic and clinical characteristics are in Supplemental Tables 1–4; supplemental material available online with this article; https://doi.org/10.1172/JCI200316DS1 Our results showed that patients with vitiligo had a higher incidence of hyperglycemia than healthy controls (11.84% vs. 7.01%, *P* = 0.012) (Figure 1A and Supplemental Table 5). After adjusting for potential confounders including age, body mass index (BMI), sex, smoking, and drinking, bivariate logistic regression analysis showed that hyperglycemia was notably positively correlated with vitiligo (adjusted odds ratio [OR] = 1.781, 95% confidence interval [CI] = 1.131–2.806, *P* = 0.013). Moreover, patients with progressive vitiligo had a substantially higher incidence of hyperglycemia than those with stable vitiligo (15.02% vs. 5.56%, *P* = 0.013) (Figure 1B and Supplemental Table 6). Following adjustment for confounding factors, bivariate logistic regression analysis showed that hyperglycemia was remarkably positively correlated with vitiligo activity (adjusted OR = 3.006, 95% CI = 1.216–7.430, *P* = 0.017). Additionally, there was a significant difference in the incidence of hyperglycemia among different groups of vitiligo severity (3.36% vs. 15.92% vs. 20.00, *P* < 0.001) (Figure 1C and Supplemental Table 7). After adjustment for confounding factors, ordinal multivariable logistic regression analysis revealed that hyperglycemia was markedly associated with vitiligo severity (adjusted OR = 2.707, 95% CI = 1.306–5.609, *P* = 0.007). However, no significant difference was found in the incidence of hyperglycemia between segmental and nonsegmental patients with vitiligo (5.56% vs. 13.00%, *P* = 0.198) (Supplemental Figure 1A and Supplemental Table 8). The undetermined vitiligo group (*n* = 8) was excluded from this analysis as it contained no cases with hyperglycemia. These findings highlight hyperglycemia as a potential risk factor for the development and progression of vitiligo.

To further clarify the role of hyperglycemia in the development of vitiligo in vivo, we employed a melanoma-Treg-induced vitiligo mouse model that recapitulated the pathological characteristics of CD8^+^ T cell–mediated melanocyte destruction in human vitiligo as previously described (32–34). Following the treatment with vitiligo induction, a high-fat diet combined with 100 mg/kg streptozotocin (STZ) intraperitoneal injection was applied to induce hyperglycemia (Figure 1D). Compared with control and vitiligo mice, strongly elevated glucose levels were observed in hyperglycemia-treated vitiligo mice after hyperglycemia induction (Supplemental Figure 1B). After 10 weeks of hyperglycemia, we analyzed and quantified the depigmented area on the tails in each group. As shown, the total depigmented area on the tails of hyperglycemia-treated vitiligo mice was substantially larger than that in vitiligo mice (Figure 1E and Supplemental Figure 1, C and D). Importantly, depigmented areas showed a positive correlation with glucose levels at week 10 (Figure 1F). Progressive vitiligo lesions are characterized by focal CD8^+^ T cell infiltration and melanocyte destruction in the skin (1, 35, 36). Whole-mount immunofluorescence staining and flow cytometric analysis revealed that hyperglycemia-treated vitiligo mice exhibited more extensive melanocyte loss accompanied by increased CD8^+^ T cell infiltration compared with vitiligo mice without hyperglycemia (Figure 1, G and H, and Supplemental Figure 1, E and F). Taken together, hyperglycemia might aggravate the progression of vitiligo by increasing the infiltration of CD8^+^ T cells in skin.

### Succinate is the key metabolite associated with hyperglycemia-aggravated vitiligo.

**Figure 2:**
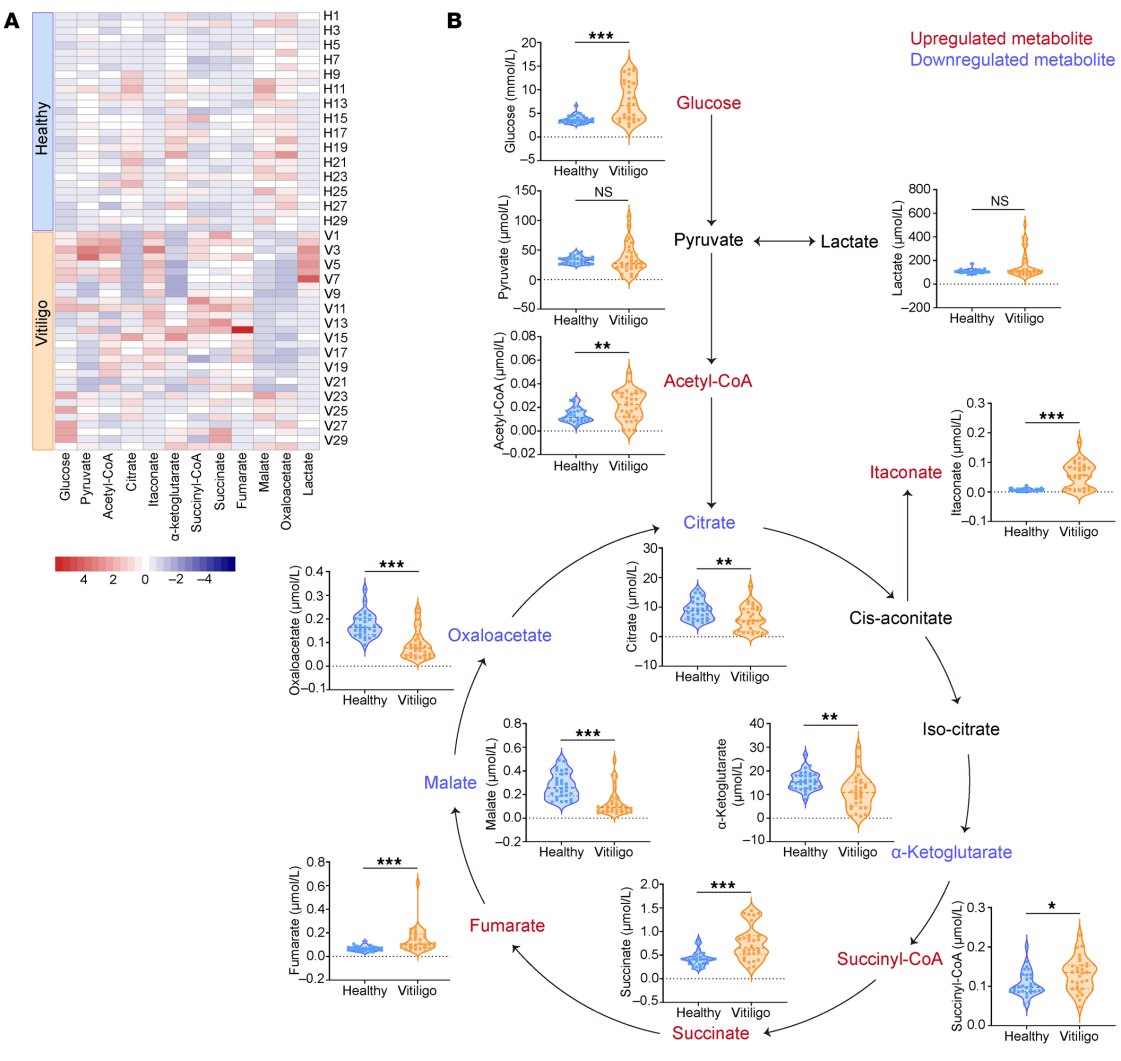
Altered serum levels of glucose-related metabolites in patients with vitiligo. (**A**) Heatmap of normalized metabolite abundance in serum samples from vitiligo patients and healthy controls (*n* = 30). (**B**) Violin plots showing individuals’ indicated metabolism levels in each group. Data are presented as median ± IQR and analyzed by Mann-Whitney *U* test (**B**). *P < 0.05; **P < 0.01; ***P < 0.001.

**Figure 3:**
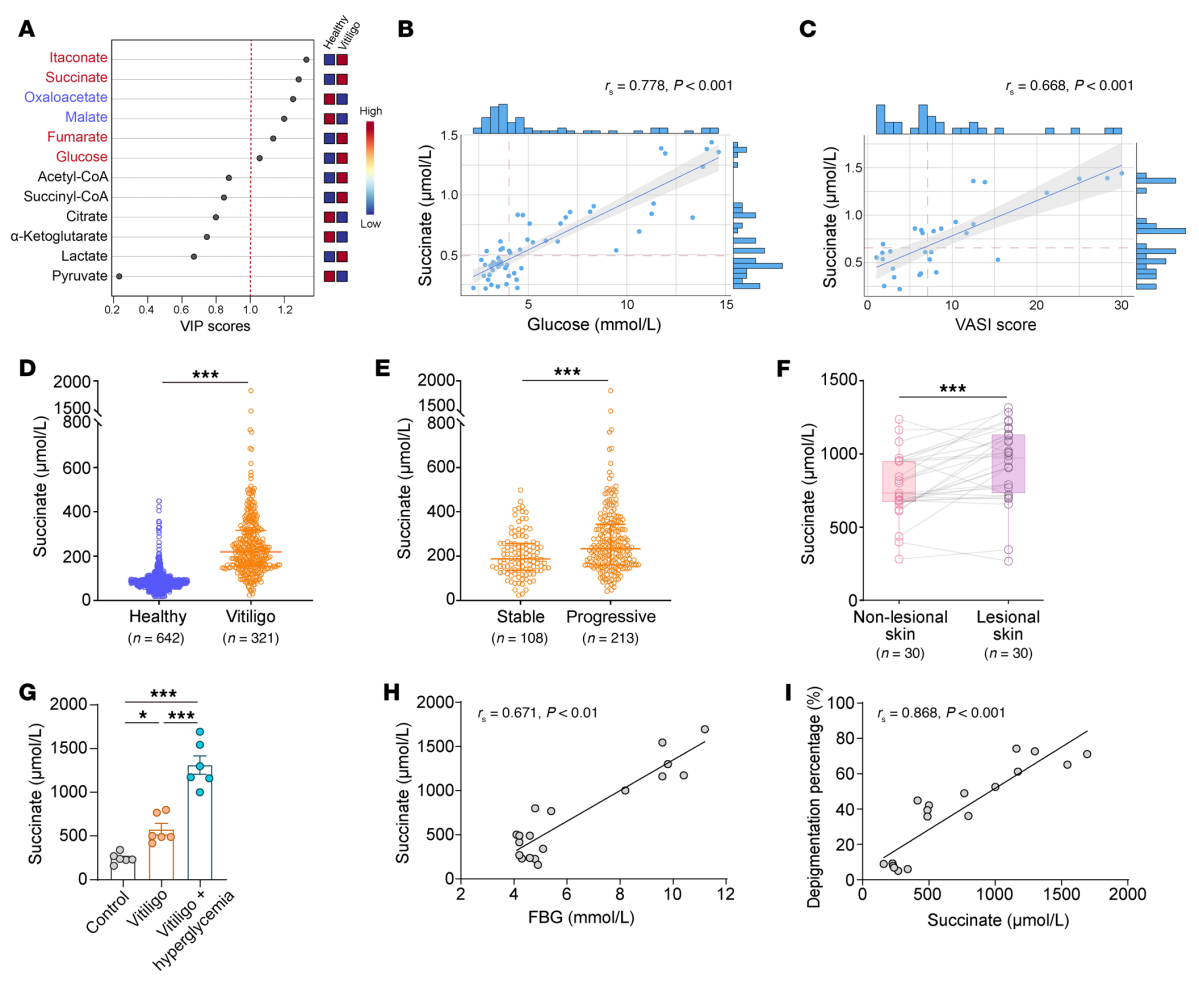
Succinate is the potential metabolite mediating the exacerbation of vitiligo by hyperglycemia. (**A**) VIP scores of indicated metabolites. The colored boxes on the right indicate the relative concentrations of the corresponding metabolite. (**B**) Correlation between serum glucose and succinate concentration of vitiligo patients and healthy controls (*n* = 60). (**C**) Correlation between VASI scores and serum succinate concentration of vitiligo patients (*n* = 30). (**D** and **E**) Serum succinate levels of vitiligo patients and healthy controls (**D**) and vitiligo patients with different activity (**E**). (**F**) The level of succinate in blister fluid from lesional and nonlesional skin areas of vitiligo patients. (**G**) Serum succinate levels of mice in each group (*n* = 6). (**H**) Correlation between FBG and serum succinate concentration of mice at week 10 (*n* = 18). (**I**) The correlation between serum succinate concentrations and the tail depigmentation percentage of mice at week 10 (*n* = 18). Data are presented as median ± IQR (**D**, **E**, and **F**) or mean ± SEM (**G**). Data are analyzed by Mann-Whitney *U* test (**D** and **E**), Wilcoxon’s signed-rank test (**F**), or 1-way ANOVA (**G**). *P < 0.05, ***P < 0.001. VIP, variable importance in projection; VASI, vitiligo area scoring index; FBG, fasting blood glucose.

To identify the metabolite that mediates hyperglycemia-induced vitiligo progression, we performed targeted metabolomic analyses on serum samples from 30 patients with vitiligo and 30 age- and sex-matched healthy individuals. The individuals were comparable in BMI, smoking, and drinking (Supplemental Table 9). Levels of glucose and 11 TCA cycle intermediates including pyruvate, acetyl-CoA, citrate, itaconate, α-ketoglutarate, succinyl-CoA, succinate, fumarate, malate, oxaloacetate, and lactate were measured. The DModX test plot indicated none of the samples exhibited a severe deviation (Supplemental Figure 2A). As shown by orthogonal partial least squares discriminant analysis, there was a significant distinction between the 2 groups (*Q*^2^ = 0.659) (Supplemental Figure 2B). The heatmap showed substantial changes in the metabolic level in the vitiligo group (Figure 2A). Of these, apart from glucose, the levels of acetyl-CoA, itaconate, succinyl-CoA, succinate, and fumarate were increased in the serum of patients with vitiligo, while those of citrate, α-ketoglutarate, malate, and oxaloacetate were decreased (Figure 2B). Among the upregulated metabolites, glucose, itaconate, succinate, and fumarate showed VIP > 1 (indicating higher difference compared with controls), while downregulated counterparts were oxaloacetate and malate (Figure 3A). Correlation analysis showed that glucose was positively correlated with succinate, itaconate, and fumarate; no statistically significant correlation was observed between glucose and oxaloacetate, nor with malate (Figure 3B and Supplemental Figure 3A). Itaconate and fumarate are well-established immunosuppressive metabolites (31, 37–39). Notably, under conditions of immune activation, itaconate and fumarate tend to be just compensatively upregulated to balance the immune response (37). Further correlation analysis showed a positive correlation between succinate and assessed Vitiligo Area Scoring Index (VASI) scores of vitiligo (Figure 3C). Therefore, we further examined the implication of succinate in hyperglycemia-mediated vitiligo progression. Total succinate levels in the serum samples from the case-control study were measured using a succinate assay kit. As a result, serum succinate levels of patients with vitiligo were markedly higher than those of healthy controls (219.00 μmol/L vs. 80.95 μmol/L, *P* = 0.0001) (Figure 3D). Concordantly, higher succinate levels were found in progressive vitiligo (Figure 3E), serum succinate levels in moderate vitiligo were higher than those in mild vitiligo (Supplemental Figure 3B), and there were no differences between the vitiligo types (Supplemental Figure 3C). Moreover, succinate was strikingly elevated in lesional blister fluid compared with nonlesional blister fluid in patients with vitiligo (Figure 3F). Sequential in vivo experiments showed that circulating succinate levels were considerably higher in hyperglycemic vitiligo mice than in vitiligo mice (Figure 3G). A positive correlation between serum glucose and succinate levels was observed (Figure 3H). Moreover, we found a notable positive correlation between serum succinate levels and depigmentation (Figure 3I). The above results suggest that succinate may serve as a key metabolite mediating hyperglycemia-induced vitiligo progression.

### SUCNR1 mediates succinate-induced overactivation of CD8^+^ T cells in hyperglycemia.

**Figure 4:**
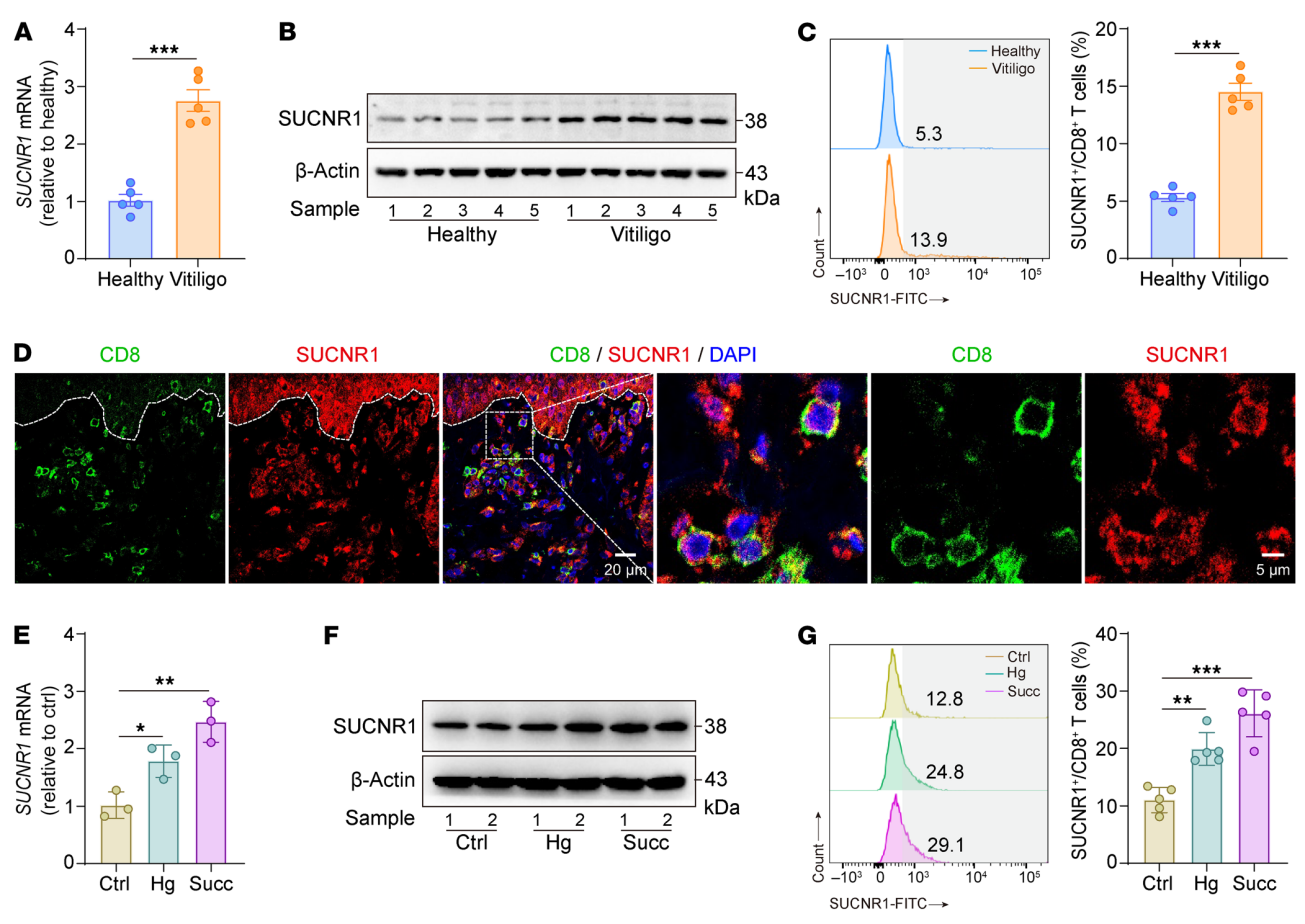
Upregulated SUCNR1 expression in vitiligo CD8^+^ T cells is modulated by hyperglycemia and succinate. (**A** and **B**) The mRNA (**A**) and protein (**B**) levels of SUCNR1 in CD8^+^ T cells from vitiligo patients and healthy controls. (**C**) Proportion of SUCNR1^+^ cells in CD8^+^ T cells of vitiligo patients and healthy controls (*n* = 5). (**D**) Representative immunofluorescence images for SUCNR1 (red) in CD8^+^ T cells in perilesional skin of vitiligo patients. CD8^+^ T cells were characterized by CD8 (green); nuclei were counterstained with DAPI (blue). (**E** and **F**) The mRNA (**E**) and protein expression (**F**) levels of SUCNR1 in CD8^+^ T cells of vitiligo patients treated with high glucose or succinate. (**G**) Proportion of SUCNR1^+^ cells in CD8^+^ T cells of vitiligo patients (*n* = 5) treated with high glucose or succinate. Data are presented as mean ± SEM (**A**, **C**, **E**, and **G**) and analyzed by 2-tailed Student’s *t* test (**A** and **C**) or 1-way ANOVA (**E** and **G**). *P < 0.05; **P < 0.01; ***P < 0.001. Ctrl, control; Hg, high glucose; Succ, succinate.

**Figure 5:**
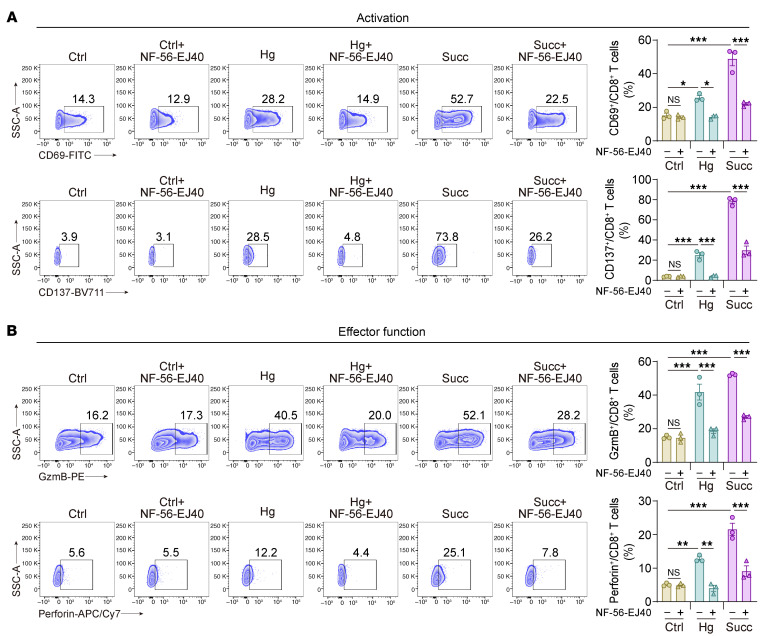
SUCNR1 mediates succinate-induced activation of CD8^+^ T cells in hyperglycemia. (**A** and **B**) Proportion of CD69^+^, CD137^+^ (**A**), granzyme B^+^, and perforin^+^ cells (**B**) in CD8^+^ T cells of vitiligo patients pretreated with NF-56-EJ40 before high-glucose or succinate treatment. Data are presented as mean ± SEM and analyzed by 1-way ANOVA (**A** and **B**). *P < 0.05; **P < 0.01; ***P < 0.001. Ctrl, control; Hg, high glucose; Succ, succinate.

We further explored how succinate promotes the CD8^+^ T cell–mediated immune response. First, we analyzed published single-cell RNA-seq data (40) and found that SUCNR1 expression was markedly higher in circulating CD8^+^ T cells from patients with vitiligo compared with those from healthy controls (Supplemental Figure 4A). qRT-PCR, Western blot, and flow cytometric analyses confirmed that the expression of SUCNR1 in CD8^+^ T cells isolated from the peripheral blood of patients with vitiligo was higher than that in healthy controls (Figure 4, A–C, and Supplemental Figure 4B). Moreover, significant SUCNR1 expression was evident in the CD8^+^ T cells infiltrating the lesions of patients with vitiligo, as demonstrated by immunofluorescence analysis (Figure 4D). Importantly, both high glucose and succinate considerably upregulated SUCNR1 expression in CD8^+^ T cells derived from patients with vitiligo (Figure 4, E–G, and Supplemental Figure 4C). Further flow cytometry analysis showed that CD8^+^ T cell activation was enhanced by high-glucose or succinate treatment, which could be attenuated by SUCNR1-specific antagonist NF-56-EJ40 (Figure 5A and Supplemental Figure 5A). Similar results were observed in effector function molecules of CD8^+^ T cells, including granzyme B and perforin (Figure 5B and Supplemental Figure 5B). Collectively, these findings suggest that SUCNR1 is the receptor through which high glucose and succinate mediate enhanced activation of CD8^+^ T cells.

### Succinate induces CD8^+^ T cells’ hyperactivation and accelerated depigmentation through SUCNR1.

**Figure 6:**
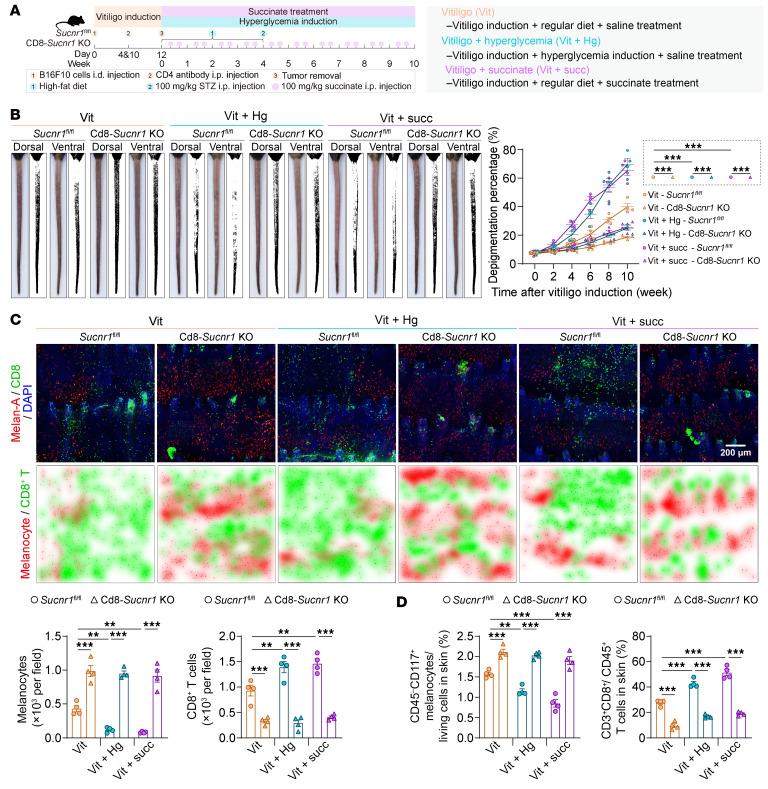
Succinate aggravates vitiligo through SUCNR1. (**A**) Schematic presentation of vitiligo, hyperglycemia, and succinate-treated mouse model establishment. Thus, 3 groups of mice (Vitiligo, Vitiligo + Hyperglycemia, Vitiligo + Succinate) were studied here. (**B**) Representative tail skin images of mice in each group at week 10 and the tail pigmentation percentages of mice in each group (*n* = 4) in 10 consecutive weeks. (**C**) Representative whole-mount immunofluorescence staining images (upper) and corresponding heatmaps (low) of melanocytes (red) and CD8^+^ T cells (green) in the tail skin epidermis of mice in each group. Numbers of melanocytes and CD8^+^ T cells in each group (*n* = 4) are shown. (**D**) Quantification of CD45^–^CD117^+^ melanocytes and CD3^+^CD8^+^ T cells in tail epidermis of mice in each group (*n* = 4). Data are presented as mean ± SEM and analyzed by 1-way ANOVA (**B**–**D**). **P < 0.01; ***P < 0.001. Vit, vitiligo; Hg, hyperglycemia; Succ, succinate.

To further clarify the role of SUCNR1 in mediating the regulation of CD8^+^ T cell immune responses influenced by hyperglycemia and succinate, we employed *Sucnr1*^fl/fl^ *Cd8*-Cre^+/–^ mice (referred to as Cd8-*Sucnr1*-KO mice), in which *Sucnr1* was knocked out in CD8^+^ T cells (Supplemental Figure 6, A and B). As expected, SUCNR1 expression was strongly reduced in blood and lymph node CD8^+^ T cells of Cd8-*Sucnr1*-KO mice when compared with *Sucnr1*^fl/fl^ mice, while no changes were observed in other tissues, such as the kidney and liver (Supplemental Figure 6C). Following vitiligo modeling, mice underwent hyperglycemia induction or succinate treatment (Figure 6A). The total depigmented area on the tail was drastically larger in both hyperglycemia- and succinate-treated vitiligo mice compared with vitiligo mice. In contrast, Cd8-*Sucnr1*-KO mice exhibited an evidently smaller tail depigmented area than *Sucnr1*^fl/fl^ control mice (Figure 6B and Supplemental Figure 7A). In addition, whole-mount immunofluorescence staining and flow cytometric analysis showed that Cd8-*Sucnr1*-KO mice exhibited markedly attenuated CD8^+^ T cell infiltration and melanocyte destruction compared with *Sucnr1*^fl/fl^ mice (Figure 6, C and D, and Supplemental Figure 7, B and C). The results above suggest that hyperglycemia and succinate regulate the progression of vitiligo through SUCNR1.

**Figure 7:**
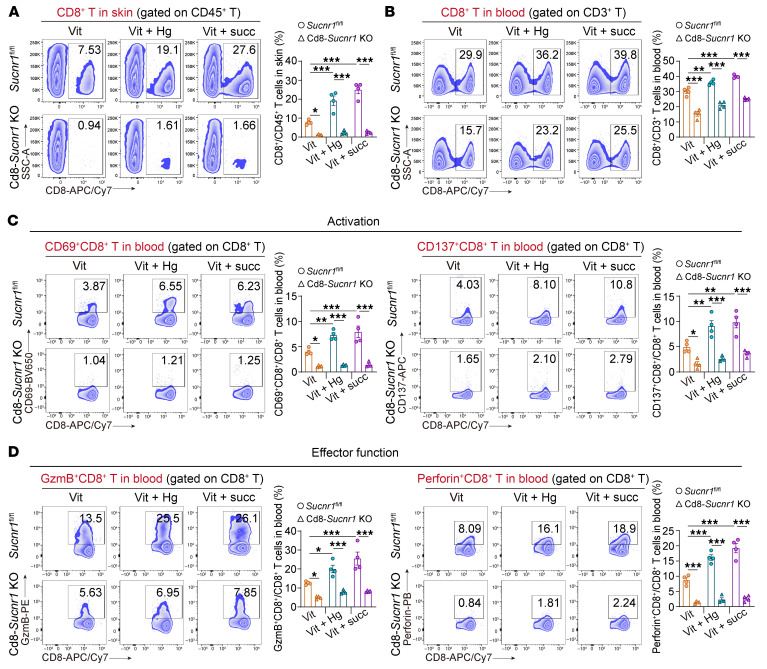
Succinate promotes hyperactivation of CD8^+^ T cells via SUCNR1. (**A**) Proportion of CD8^+^ T cells in the tail skin of mice in each group (*n* = 4). (**B**) Proportion of CD8^+^ T cells in the blood of mice in each group (*n* = 4). (**C** and **D**) Proportion of CD69^+^CD8^+^ T cells, CD137^+^CD8^+^ T cells (**C**), granzyme B^+^CD8^+^ T cells, and perforin^+^CD8^+^ T cells (**D**) in the blood of mice in each group (*n* = 4). Data are presented as mean ± SEM and analyzed by 1-way ANOVA (**A**–**D**). *P < 0.05; **P < 0.01; ***P < 0.001. Vit, vitiligo; Hg, hyperglycemia; Succ, succinate.

We further evaluated the effects of hyperglycemia and succinate on CD8^+^ T cell–mediated immune response in vivo. Consistent with previous results, knockdown of SUCNR1 in CD8^+^ T cells markedly ameliorated their skin infiltration and peripheral accumulation in vitiligo mice under hyperglycemic conditions or following succinate treatment (Figure 7, A and B, and Supplemental Figure 8A). Similar results were obtained in the primary draining lymph nodes of mouse tail skin (Supplemental Figure 8, B and C). Sequentially, CD8^+^ T cell activation and effector function were substantially increased in the hyperglycemia and succinate group in peripheral blood and lymph node, and the activation and cytotoxicity were inhibited considerably in CD8^+^ T cells from the Cd8-*Sucnr1*-KO mice compared with *Sucnr1*^fl/fl^ mice (Figure 7, C and D, and Supplemental Figure 9), though the phenomenon was not observed in tail skin (Supplemental Figure 10). Overall, these results demonstrate that hyperglycemia and succinate lead to increased number, activation, and effector function of CD8^+^ T cells by binding to SUCNR1 on CD8^+^ T cells.

### Succinate-induced chemokine secretion via SUCNR1 in keratinocytes promotes CD8^+^ T cell migration.

**Figure 8:**
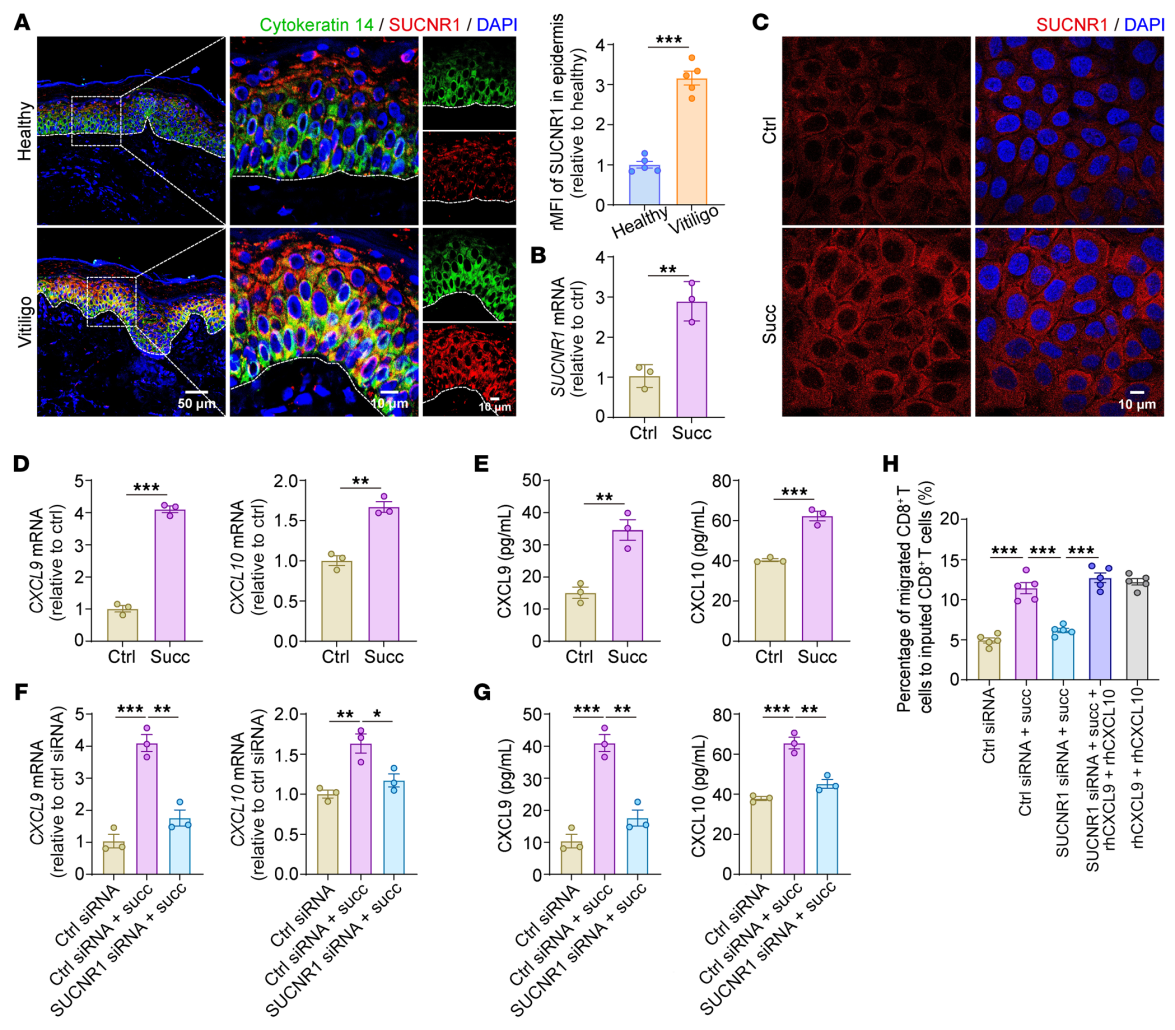
Succinate-induced chemokine secretion via SUCNR1 in keratinocytes promotes CD8^+^ T cell migration. (**A**) Representative immunofluorescence images and quantification of SUCNR1 (red) in keratinocytes from vitiligo patients and healthy controls (*n* = 5). Keratinocytes were characterized by cytokeratin 14 (green); nuclei were counterstained with DAPI (blue). Scale bars: 50 μm (left), 10 μm (middle and right). (**B**) The mRNA level of SUCNR1 in NHKs treated with succinate. (**C**) Representative images of SUCNR1 expression in succinate-treated NHKs. (**D**) The mRNA levels of *CXCL9* and *CXCL10* in NHKs treated with succinate. (**E**) The secretion of CXCL9 and CXCL10 in NHKs treated with succinate. (**F** and **G**) The mRNA (**F**) and secretion (**G**) levels of CXCL9 and CXCL10 in NHKs treated with succinate or pretreated with SUCNR1 siRNA prior to succinate stimulation. (**H**) Transwell assay showing the percentage of migrated CD8^+^ T cells in response to culture supernatants from succinate-treated NHKs with knockdown of SUCNR1 or with the addition of rhCXCL9 or rhCXCL10 into the Transwell system. Data are presented as mean ± SEM (**A**, **B**, and **D**–**H**) and analyzed by Student’s *t* test (**A**, **B**, **D**, and **E**) or 1-way ANOVA (**F**–**H**). *P < 0.05, **P < 0.01, ***P < 0.001. NHKs, normal human keratinocytes; Ctrl, control; Succ, succinate; rhCXCL9, recombinant human CXCL9; rhCXCL10, recombinant human CXCL10; rMFI, relative mean fluorescence intensity.

As shown in Figure 3F, blister fluid from vitiligo lesional skin contained higher levels of succinate than that from nonlesional skin, indicating that elevated succinate in the skin microenvironment contributes to vitiligo development. Moreover, we found that SUCNR1 expression levels were increased in lesional keratinocytes of patients with vitiligo than those in healthy controls based on published single-cell RNA-Seq data (32) (Supplemental Figure 11A). To confirm this finding at the protein level, we performed immunofluorescence analysis, demonstrating increased SUCNR1 expression in keratinocytes of patients with vitiligo (Figure 8A). In addition, succinate treatment markedly elevated the mRNA and protein levels of SUCNR1 in normal human keratinocytes (NHKs) (Figure 8B and Supplemental Figure 11B). Immunofluorescence analysis confirmed the upregulation of SUCNR1 in succinate-treated NHKs (Figure 8C). Studies have illustrated that keratinocyte-derived chemokines CXCL9, CXCL10, and CXCL16 recruit CD8^+^ T cells to the epidermis to kill melanocytes in vitiligo (41, 42). Our results showed that, compared with control, the mRNA and secretion levels of CXCL9 and CXCL10 were increased in succinate-treated NHKs (Figure 8, D and E), while there were no significant changes in the mRNA and secretion levels of CXCL16 (Supplemental Figure 11, C and D). Increased expression of CXCL9 and CXCL10 was observed in keratinocytes of succinate-treated vitiligo mice by immunofluorescence analysis (Supplemental Figure 11E). Notably, pretreatment with SUCNR1 siRNA or SUCNR1 antagonist NF-56-EJ40 markedly dampened succinate-induced increase of CXCL9 and CXCL10 in both mRNA and protein levels (Figure 8, F and G, and Supplemental Figure 11, F and G). Transwell assay showed that the medium from succinate-treated NHKs facilitated migration of vitiligo patient–derived CD8^+^ T cells, which could be attenuated by either knockdown or inhibition of SUCNR1 in NHKs but reversed by recombinant human CXCL9 or CXCL10 supplement (Figure 8H and Supplemental Figure 11H). Our findings demonstrate that keratinocyte SUCNR1-mediated secretion of CXCL9 and CXCL10 under succinate promotes CD8^+^ T cell migration.

### Succinate stabilizes HIF-1α through SUCNR1 to upregulate CXCL9 and CXCL10.

**Figure 9:**
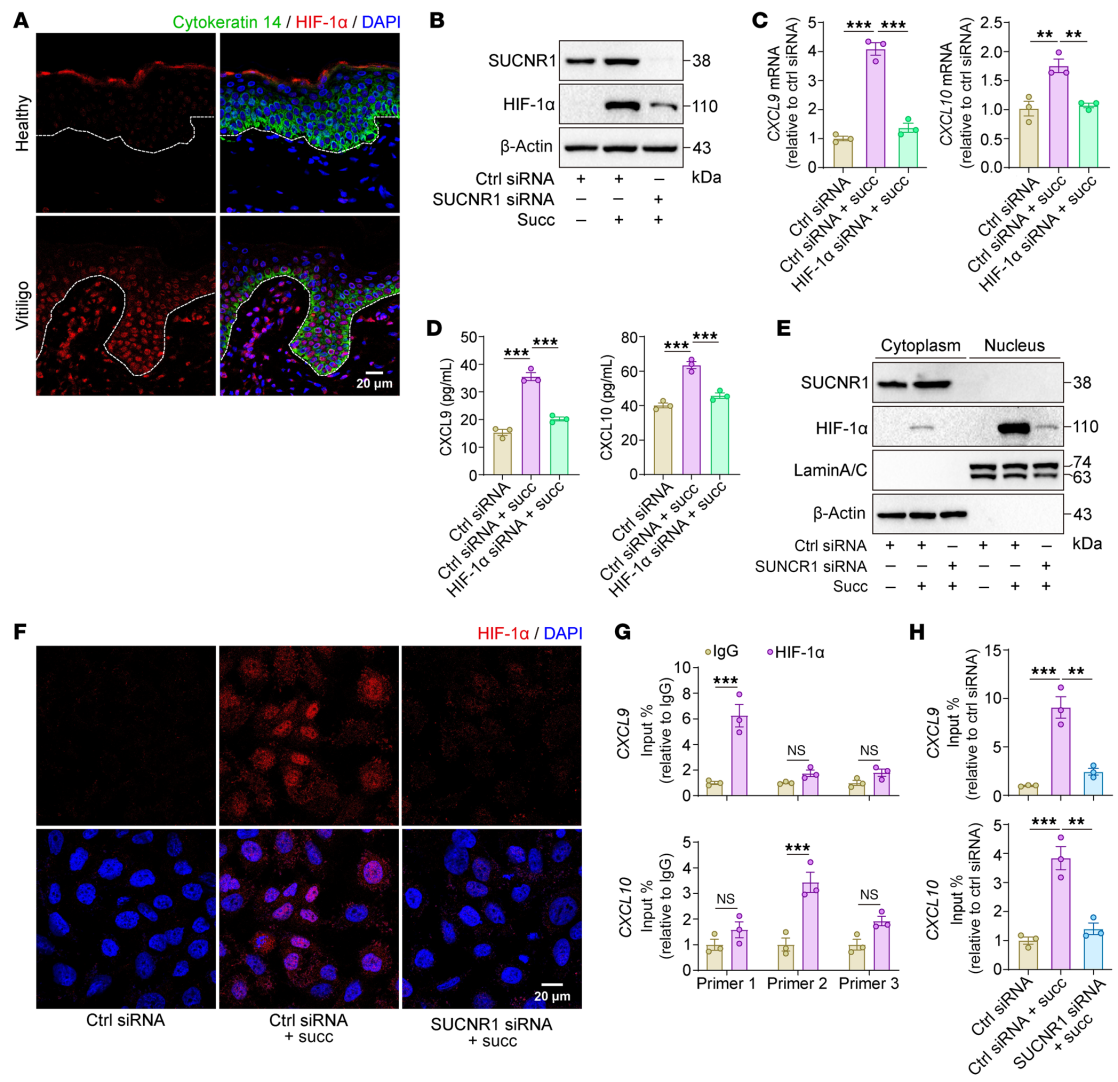
Succinate promotes HIF-1α nuclear translocation and its binding to CXCL9/CXCL10 promoters in keratinocytes via SUCNR1. (**A**) The expression of HIF-1α (red) in keratinocytes in vitiligo patients and healthy controls. Keratinocytes were characterized by cytokeratin 14 (green); nuclei were counterstained with DAPI (blue). (**B**) Protein levels of SUCNR1 and HIF-1α in NHKs pretransfected with SUCNR1 siRNA prior to succinate stimulation. (**C** and **D**) The mRNA (**C**) and secretion (**D**) levels of CXCL9 and CXCL10 in NHKs pretransfected with HIF-1α siRNA prior to succinate stimulation. (**E**) The expression of SUCNR1 and HIF-1α in the cytoplasm and nucleus of NHKs pretransfected with HIF-1α siRNA prior to succinate stimulation. (**F**) The expression of HIF-1α in NHKs pretransfected with SUCNR1 siRNA prior to succinate stimulation. (**G**) Binding of HIF-1α to *CXCL9* and *CXCL10* promoter regions in NHKs. (**H**) Binding of HIF-1α to *CXCL9* and *CXCL10* promoter region in NHKs pretreated with SUCNR1 siRNA prior to succinate treatment. Data are presented as mean ± SEM (**C**, **D**, **G**, and **H**) and analyzed by 1-way ANOVA (**C**, **D**, and **H**) or 2-tailed Student’s *t* test (**G**). **P < 0.01; ***P < 0.001; NHKs, normal human keratinocytes; Ctrl, control; Succ, succinate.

**Figure 10:**
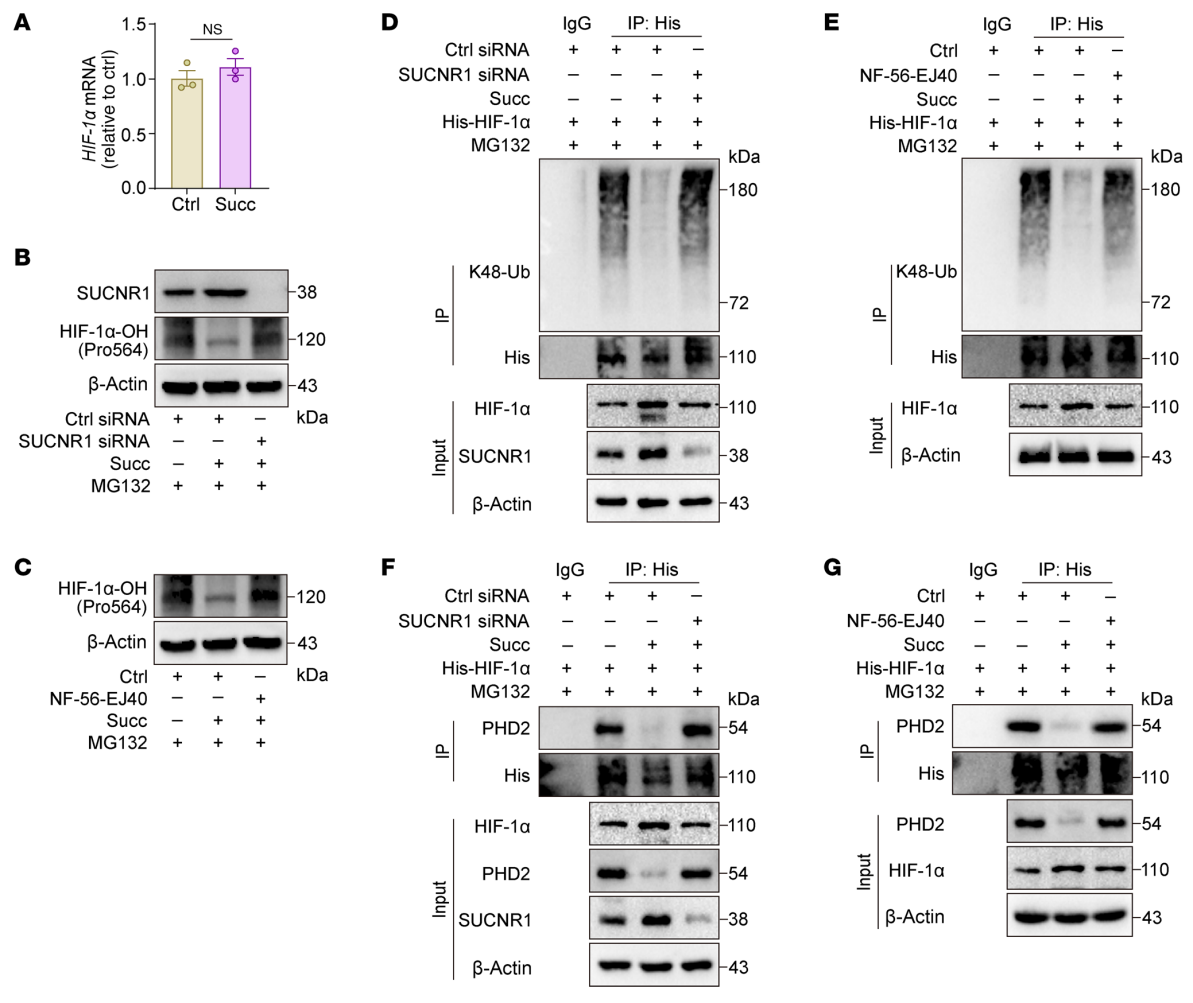
Succinate enhances HIF-1α stability through SUCNR1. (**A**) The mRNA level of HIF-1α in NHKs treated with succinate. (**B** and **C**) Expression of HIF-1α-OH in NHKs pretreated with SUCNR1 siRNA (**B**) or NF-56-EJ40 (**C**) prior to succinate treatment. (**D** and **E**) The ubiquitination of HIF-1α in NHKs pretreated with SUCNR1 siRNA (**D**) or NF-56-EJ40 (**E**) prior to succinate treatment. (**F** and **G**) The binding of HIF-1α and PHD2 in NHKs pretreated with SUCNR1 siRNA (**F**) or NF-56-EJ40 (**G**) prior to succinate treatment. Data are presented as mean ± SEM and analyzed by 2-tailed Student’s *t* test (**A**). NHKs, normal human keratinocytes; Ctrl, control; Succ, succinate.

Next, we investigated the mechanisms by which succinate induces the secretion of CXCL9 and CXCL10 in NHKs. Hypoxia-inducible factor-1α (HIF-1α) is well known as a hallmark of hypoxia and has been shown to mediate the secretion of pro-inflammatory cytokines induced by the succinate/SUCNR1 axis, such as IL-1β (43, 44). As shown by immunofluorescence, the expression of HIF-1α was upregulated in keratinocytes from vitiligo lesions versus in those from healthy skin (Figure 9A). Western blot assay confirmed that succinate markedly increased the expression of HIF-1α, and both SUCNR1 siRNA and NF-56-EJ40 pretreatment reversed succinate-induced HIF-1α upregulation (Figure 9B and Supplemental Figure 12A). Importantly, HIF-1α siRNA pretreatment could ameliorate the elevated CXCL9 and CXCL10 transcription and secretion levels in succinate-stimulated NHKs (Figure 9, C and D). To further explore the mechanism underlying the regulation of CXCL9 and CXCL10, as shown by Western blot assay, the strongly increased expression of HIF-1α was mainly observed in the cell nucleus; both SUCNR1 siRNA and NF-56-EJ40 decreased HIF-1α expression in the cell nucleus (Figure 9E and Supplemental Figure 12B). Immunofluorescence analysis also showed that both SUCNR1 siRNA and NF-56-EJ40 inhibited the succinate-induced nuclear translocation of HIF-1α in NHKs (Figure 9F and Supplemental Figure 12C). Studies have shown that nuclear localized HIF-1α functions primarily as a transcription factor to upregulate hypoxia-responsive genes, including chemokines such as CCL2 and CXCL8 (45, 46). To further investigate whether HIF-1α directly regulates the transcription of CXCL9 and CXCL10, we screened the JASPAR database (http://jaspar.genereg.net), analyzed the –2,000 to +1 region of the *CXCL9* and *CXCL10* promoter sequences, and identified 6 and 3 putative HIF-1α–responsive elements, respectively (Supplemental Figure 12D). ChIP assay showed that HIF-1α binds to the first predicted binding site in the promoter region of *CXCL9* and the second predicted binding site in the promoter region of *CXCL10* (Figure 9G). Notably, our results indicate that SUCNR1 siRNA or NF-56-EJ40 diminished the binding of HIF-1α to the *CXCL9* and *CXCL10* promoter regions induced by succinate (Figure 9H and Supplemental Figure 12E). As shown in Figure 9B and Supplemental Figure 12A, succinate promoted the expression of HIF-1α in NHKs, while it had no significant effect on HIF-1α transcription levels (Figure 10A). HIF-1α is a well-known proline hydroxylation substrate, and the modification of HIF-1α by prolyl hydroxylase domain-containing protein 2 (PHD2) tightly regulates the protein level of HIF-1α in the cells by regulating its ubiquitination and proteasome-dependent protein degradation (47). Thus, we investigated whether succinate stabilizes HIF-1α by inhibiting its prolyl hydroxylation and ubiquitination by SUCNR1. Western blot and co-immunoprecipitation (co-IP) assays showed that knockdown or inhibition of SUCNR1 can reverse the decrease of prolyl hydroxylation and concomitantly ubiquitination of HIF-1α induced by succinate (Figure 10, B–E). Moreover, co-IP assays demonstrated that succinate markedly decreased the expression of PHD2 and the interaction of HIF-1α with PHD2 in NHKs; knockdown or inhibition of SUCNR1 reversed the conditions (Figure 10, F and G). Taken together, these findings suggest that succinate decreased the interaction of HIF-1α with PHD2 via SUCNR1, preventing its prolyl hydroxylation, ubiquitination, and degradation and thus increasing its transcriptional activity, which promotes the transcription of CXCL9 and CXCL10.

## Discussion

This study uncovered the molecular mechanism by which hyperglycemia promoted the progression of vitiligo through an immune-metabolic axis mediated by the metabolic intermediate succinate. We found that hyperglycemia leads to the accumulation of succinate, which exerts dual functional roles by binding to its receptor SUCNR1. On the one hand, succinate augmented the activation and effector functions of CD8^+^ T cells. On the other hand, succinate-SUCNR1 interaction stabilized HIF-1α by inhibiting its prolyl hydroxylation and ubiquitination, thereby enhancing its transcriptional activity and markedly upregulating the expression of the chemokines CXCL9 and CXCL10 in keratinocytes. This dual mechanism exacerbated the recruitment of hyperactivated CD8^+^ T cells and the concomitant melanocyte destruction. These findings establish the hyperglycemia/succinate/SUCNR1 signaling axis as a key regulator of hyperglycemia-associated vitiligo progression. This work provides a conceptual framework for understanding metabolism-immune crosstalk in vitiligo and highlights potential therapeutic targets for vitiligo patients with comorbid hyperglycemia.

Our case-control analysis confirmed that hyperglycemia is a risk factor for hyperglycemia-complicated vitiligo, and is positively correlated with disease activity and severity. These results are aligned with our previous meta-analysis and corroborate another study that reported associations between hyperglycemia and vitiligo (10, 11). However, a study conducted in Egypt (48) reported no significant difference in blood glucose levels between patients with vitiligo and healthy controls. The discrepancy may be attributed to differences in genetic background, a small sample size, or methodological limitations, such as failing to distinguish between fasting and postprandial glucose levels. In our study, we accounted for potential confounders, such as BMI and smoking status, and utilized a larger sample size, enabling more robust identification of hyperglycemia as a risk factor for vitiligo onset and progression. Additionally, we employed clinically defined hyperglycemia status rather than continuous glucose variables, as categorical variables directly reflecting disease diagnosis are more reliable than quantitative metrics that indirectly infer pathological states (10). Notably, the present study demonstrated that hyperglycemia evidently accelerated the expansion of depigmented lesions utilizing a vitiligo mouse model, and the white area was strongly correlated with glucose levels. These findings underscore the clinical imperative for regular glycemic monitoring in patients with vitiligo, particularly during active phases, and suggest that early metabolic interventions may mitigate disease progression, especially in patients with concurrent glucose metabolism disorders.

Succinate is a key intermediary metabolite in the TCA cycle, serving dual roles as an energy substrate and an immune regulation signaling molecule functioning in various pathological conditions, like inflammatory bowel disease, ischemia/reperfusion injury, and cancer (49). In the present study, we applied targeted metabolomics to quantify serum succinate concentrations in large-scale population samples. Our results demonstrate that serum succinate levels are substantially elevated in individuals with vitiligo relative to the general population, which was consistent with a previous study (21). Furthermore, serum succinate levels show a positive correlation with vitiligo disease activity, severity, and blood glucose levels. As for the biomarker, the estimation of vitiligo activity mainly relies on visible depigmentation changes, which lag behind actual disease activity by 2–3 months due to the epidermal turnover time (50). The correlation of serum succinate with both VASI scores and lesional area supports its potential as an early and objective biomarker for disease severity. Our findings align with prior studies (51), demonstrating succinate elevation as a hallmark of inflammatory conditions, further emphasizing its potential as a broadly applicable inflammatory biomarker. Nevertheless, comprehensive assessments of its sensitivity and specificity across heterogeneous clinical phenotypes are needed to define its diagnostic precision.

In the context of vitiligo, we unveil the dual functional role of SUCNR1 in both CD8^+^ T cells and keratinocytes. In CD8^+^ T cells, using pharmacological inhibition and genetic manipulation, we found that succinate binding to SUCNR1 increased the proportions of granzyme B^+^ and perforin^+^CD8^+^ T cells, thereby increasing their capacity to kill melanocytes. Interestingly, previous studies in tumor immunology have revealed that succinate exerts a dose-dependent, dual effect on CD8^+^ T cells’ function: high concentrations of succinate in the tumor microenvironment can suppress their antitumor activity, whereas moderate levels may promote memory formation (52). This dual role highlights succinate as a key metabolic regulator of CD8^+^ T cell function across different disease contexts. In keratinocytes, succinate-SUCNR1 activation induced the production of CXCL9 and CXCL10, thereby establishing a chemokine gradient that facilitated the recruitment of CD8^+^ T cells to the epidermis. A similar pro-inflammatory effect of succinate was observed previously (53). In rheumatoid arthritis, synovial fluid succinate activates SUCNR1 to induce IL-1β secretion from macrophages, exacerbating inflammation (15). In obesity-related metabolic disorders, SUCNR1 signaling in adipose tissue macrophages drives chronic inflammation linked to type 2 diabetes (26). To conclude, the present study suggests that the succinate-SUCNR1 axis orchestrates a self-amplifying inflammatory loop between immune cells and keratinocytes, thereby driving vitiligo progression, and supports SUCNR1 as a therapeutic target in vitiligo.

CXCL9 and CXCL10 are well-characterized chemokines involved in vitiligo pathogenesis, with CXCL9 serving as an early marker of disease onset (41) and CXCL10 contributing to the maintenance of disease activity (54). While previous studies have primarily attributed their regulation to immune-mediated signaling pathways (55, 56), our findings uncover a distinct, metabolically driven mechanism. Specifically, we show that succinate stabilizes HIF-1α in keratinocytes via a noncanonical pathway that operates independently of hypoxia. By inhibiting PHD2-mediated hydroxylation, succinate prevents HIF-1α ubiquitination and degradation, thereby prolonging its nuclear retention and enhancing its transcriptional activity. In contrast with the reported mechanism where oxidative stress–induced HIF-1α promotes the adhesion of CD8^+^ T cells to melanocytes via occludin (57, 58), our study identifies an additional pathway in which metabolite-driven HIF-1α drives the transcriptional upregulation of *CXCL9* and *CXCL10*, thereby promoting CD8^+^ T cell recruitment to the skin. These results expand our understanding of how metabolic cues can shape the immune microenvironment in autoimmune skin disorders such as vitiligo, offering an additional perspective beyond classical inflammatory signaling.

In conclusion, this study enhances our understanding of the fundamental mechanisms of hyperglycemia-aggravated vitiligo, and it explains this process by exploring the hyperglycemia/succinate/SUCNR1 axis in vitiligo immune regulation through the integration of clinical data, metabolomic profiling, and mechanistic analysis. It proposes an expanded conceptual framework in which dysregulated immunometabolism should be considered an important contributor to vitiligo progression. These findings not only provide a rationale for SUCNR1-targeted therapies but also suggest that for the subgroup of vitiligo patients with comorbid hyperglycemia, interventions such as glycemic control and succinate antagonism may represent a complementary approach to addressing an unmet therapeutic need.

This study has several limitations. First, due to its case-control design, causal relationships between hyperglycemia and vitiligo progression cannot be established; prospective cohort studies are needed to track the dynamic impact of glycemic control on disease activity. Second, the STZ-induced hyperglycemia model primarily simulates acute insulin deficiency and may not fully recapitulate the immune adaptations associated with chronic metabolic dysfunction in humans. Third, while this model effectively mimics the hyperglycemia and metabolic profile of diabetes, the high-fat diet component introduces additional variables, such as dyslipidemia, which can independently influence immune responses. Future studies utilizing nonobese diabetic models or precise glucose-clamping techniques are warranted to further dissect the specific contribution of hyperglycemia in exacerbating vitiligo. Fourth, under hyperglycemic conditions, the cellular sources of succinate and its interactions with other metabolites remain to be fully elucidated. Fifth, larger multicenter studies are required to validate the biomarker potential of succinate.

## Methods

### Sex as a biological variable.

Both male and female individuals were included in the human study. For the animal experiments, female mice were exclusively used. This choice was based on the original study describing the current vitiligo induction protocol, which explicitly noted a higher success rate (proportion of mice developing epidermal depigmentation) in female mice than in male mice (33). Moreover, female mice have since been the preferred sex in subsequent studies using this model (32, 34, 59).

### Patients and clinical samples.

Patients with vitiligo and healthy volunteers in the hospital-based case-control study were recruited from the Department of Dermatology and Physical Examination Center of Xijing Hospital of the Fourth Military Medical University from 2020 to 2023. Vitiligo activity, severity, and classification were assessed using international consensus guidelines (60). Serum samples were collected from all individuals. Blister fluid was obtained from patients with vitiligo who underwent autologous transplantation of noncultured epidermal cell suspension. Details of the individuals and samples are described in Supplemental Methods.

### Generation of Cd8-specific deletion of Sucnr1 in mice.

Cd8 cell-specific Sucnr1-knockout mice (Cd8-*Sucnr1*-KO) were generated by breeding Cd8-Cre mice (C001333, Cyagen) with *Sucnr1*^fl/fl^ mice (CKOCMP-84112-Sucnr1-B6J-VA, Cyagen). The strategy used for identification of Cd8-*Sucnr1*-KO mice is illustrated in Supplemental Methods.

### Induction of vitiligo mice and treatment.

Melanoma-Treg-induced vitiligo mouse model was established as previously described (32–34). Hyperglycemia was induced via 4 weeks of high-fat diet (HF60, Dyets) combined with STZ (S0130, Sigma-Aldrich) intraperitoneal injection. The succinate treatment group received 100 mg/kg succinate (S8250, Solarbio) intraperitoneal injection twice per week. Details of the animals are described in Supplemental Methods.

### Cell culture and treatments.

NHKs were treated with succinate (S8250, Solarbio), NF-56-EJ40 (HY-130246, MedChemExpress), siRNA for SUCNR1 (sc-62407, Santa Cruz Biotechnology) and HIF-1α (sc-35561, Santa Cruz Biotechnology), and plasmids for HIF-1α (6*His, NM_001530.4, Tsingke). Details for cell culture and treatments are described in Supplemental Methods.

### PBMC and CD8^+^ T cell isolation and treatment.

PBMCs were isolated from the peripheral blood of patients with vitiligo and healthy controls using Ficoll density gradient centrifugation. CD8^+^ T cells were isolated from PBMCs using the Human CD8^+^ T Cell Isolation Kit (130-096-495, Miltenyi Biotec) according to the manufacturer’s instructions. Cells were treated with high glucose (G8150, Solarbio), succinate (S8250, Solarbio), and NF-56-EJ40 (HY-130246, MedChemExpress). Details of the experimental protocols are described in Supplemental Methods.

### Single-cell suspensions of mouse tail epidermis, mouse tail skin, peripheral blood, and lymph.

Single-cell suspension of mouse tail epidermis was obtained by grinding the epidermis after separating the epidermis from the dermis. Tail skin of mouse was digested into single-cell suspension using the Multi Tissue Dissociation Kit (130-110-201, Miltenyi Biotec). Single-cell suspension of mouse peripheral blood was obtained by removing red blood. Lymph node single-cell suspension was obtained by grinding the inguinal lymph nodes. More details of the experimental protocols are described in Supplemental Methods.

### FBG measurement.

FBG levels of the individuals in the case-control study were measured in the laboratory department of Xijing Hospital. FBG levels of mice were measured using a glucometer (Accu-Chek, Roche). Details of the experimental protocols and standards of hyperglycemia are described in Supplemental Methods.

### Succinate colorimetric assay.

Succinate levels in serum and blister fluid were detected by using a succinate assay kit (K-SUCC, Megazyme) according to the manufacturer’s instructions. Details of the experimental protocols are described in Supplemental Methods.

### Whole-mount immunostaining and imaging of mouse tail epidermis.

The infiltration of CD8^+^ T cells and the number of melanocytes in mouse tail skin were detected by whole-mount immunostaining. Details for whole-mount immunostaining and antibody information are described in Supplemental Methods.

### qRT-PCR assay.

Total RNA was extracted and reverse-transcribed into cDNA. The mRNA levels of CXCL9, CXCL10, CXCL16, and HIF-1α were measured by qRT-PCR. Details for qRT-PCR and the primer sequences are described in Supplemental Methods.

### Western blot assay.

Total protein was harvested from cells using RIPA lysis buffer (P0013C, Beyotime Biotechnology). Nuclear and cytosolic proteins were extracted using a nuclear and cytosolic protein extraction kit (P0027, Beyotime Biotechnology) according to the manufacturer’s instructions. Expression of SUCNR1, HIF-1α, and HIF-1α-OH were detected by Western blot assay. Details for Western blot assay and antibody information are described in Supplemental Methods.

### Flow cytometry.

PBMC, CD8^+^ T cell, or single-cell suspension staining was performed followed by fixation, permeabilization, and intracellular staining. The stained cells were analyzed by LSRFortessa flow cytometer (BD Biosciences). Details for flow cytometry and antibody information are described in Supplemental Methods.

### Immunofluorescence assay.

Expression of SUCNR1, HIF-1α, CXCL9, and CXCL10 in skin samples or NHKs was detected by immunofluorescence staining with the primary and secondary antibodies. Details for immunofluorescence assay and antibody information are described in Supplemental Methods.

### ELISA.

Secretion of CXCL9, CXCL10, and CXCL16 was detected by corresponding ELISA kit according to the manufacturer’s instructions. Details for ELISA are described in Supplemental Methods.

### Transwell migration assay.

Culture supernatants of NHKs with or without recombinant human CXCL9 (392-MG, R&D Systems) and CXCL10 (266-IP, R&D Systems) were used to detect the migrative ability of blood-derived CD8^+^ T cells by using a Transwell assay. More details are described in Supplemental Methods.

### ChIP assay.

Binding of HIF-1α to *CXCL9* and *CXCL10* promoter region was detected using the SimpleChIP Plus Sonication Chromatin IP Kit (56383, Cell Signaling Technology) according to the manufacturer’s instructions. Details for the experiment, promoter regions of *CXCL9* and *CXCL10*, and primer sequences are shown in Supplemental Methods.

### Co-IP assay.

HIF-1α in whole cell lysates subjected to co-IP by the appropriate primary antibody and subjected to Western blot analysis to detect PHD2 binding and ubiquitination of HIF-1α. Details for co-IP assay and antibody information are described in Supplemental Methods.

### Targeted liquid chromatography-mass spectrometry metabolomics analysis.

Levels of glucose and 11 TCA cycle intermediates including pyruvate, acetyl-CoA, citrate, itaconate, α-ketoglutarate, succinyl-CoA, succinate, fumarate, malate, oxaloacetate, and lactate were measured by using targeted metabolomic analyses. Details for targeted liquid chromatography-mass spectrometry metabolomic assay and data analyses are described in Supplemental Methods.

### Single-cell RNA-seq analysis.

SUCNR1 expression in CD8^+^ T cells from peripheral blood and skin lesions of patients with vitiligo and healthy controls was analyzed based on public single-cell RNA-seq data (accession numbers PRJCA006797 and GSE231794). Detailed analysis methods are described in Supplemental Methods.

### Statistics.

Data analysis was performed using GraphPad Prism version 9.0 software. *P* values less than 0.05 were considered statistically significant. Each experiment was independently repeated at least 3 times. Details for statistical analysis are described in Supplemental Methods.

### Study approval.

The human study was designed and executed according to the principles of the Declaration of Helsinki and the Department of Health and Human Services. The human research was approved by the Ethics Committee of the Fourth Military Medical University (No. KY20202019-C-1), and written informed consent was obtained from all participants. The study was registered (ClinicalTrials.gov) (Identifier: NCT05968235). All animal experiments complied with the Animal Research: Reporting of In Vivo Experiments guidelines and were carried out in accordance with the NIH’s *Guide for the Care and Use of Laboratory Animals* (National Academies Press, 2011). All animal studies were reviewed and approved by the Ethics Committee of Animal Care of the Fourth Military Medical University (No. 20250066).

### Data availability.

All data associated with this study are presented in the main figures and supplement. Values for all data points in graphs are reported in the Supporting Data Values file. There are no restrictions on data availability.

## Author contributions

PK, CL, and JC conceived the project and designed the experiments. PK performed all experiments, data analyses, and drafted the manuscript unless otherwise noted. YC and JC edited and revised the manuscript. ZS helped design the case-control study. TW, YW, PD, and BL assisted in participants recruitment and data collection. TW also contributed to conducting some experiments. XY and JC provided technical assistance. PK and YW obtained the funding for this study. CL and SL reviewed the manuscript. PK, YC, and TW are listed as co–first authors. The order of authorship was determined based on the relative magnitude of their contributions to the study. All authors read and approved the final version of the manuscript.

## Conflict of interest

The authors have declared that no conflict of interest exists.

## Funding support

- National Natural Science Foundation of China (No. 82273517, 82473519, and 82304005).

## Supplementary Material

Supplemental data

Unedited blot and gel images

Supporting data values

## Version 1 06/15/2026

Electronic publication
