# Supplementary material for: Hyperglycemia aggravates vitiligo through succinate/SUCNR1-mediated T cell activation
Source: J Clin Invest. 2026 Jun 15;136(12):e200316. doi: 10.1172/JCI200316 (PMC13262724; doi:10.1172/JCI200316)
Supplement: Supplemental data [file jci-136-200316-s185.pdf]

Supplemental Materials for

Hyperglycemia aggravates vitiligo through succinate/SUCNR1-mediated T cell activation

Pan Kang[1], Yuqian Chang[1], Tingting Wang[1], Xiuli Yi[1], Yinghan Wang[1], Pengran Du[1], Jiaxi Chen[1], Baizhang Li[1], Shuli Li[1], Zhongjun Shao[2], Jianru Chen[1,3], Chunying Li[1]

Correspondence: Chunying Li (lichying@fmmu.edu.cn), Jianru Chen (chenjr0516@fmmu.edu.cn)

This file includes:

1. Supplemental Tables 1 - 9
2. Supplemental Figures 1 - 12
3. Supplemental Methods

Supplemental Table 1. Baseline characteristics of the participants in the case-control study

**Characteristics**	**Healthy (n = 642)**	**Vitiligo (n = 321)**	***U*-Value/ χ^2-Value**	***p*-Value**
**Age, years, median (IQR)**	37.00 (13.00)	36.00 (14.00)	100718.500	0.568
**BMI, kg/m^2, median (IQR)**	23.64 (4.85)	23.56 (4.75)	97682.000	0.188
**Gender, n (%)**			<0.001	1.000
Male	316 (49.22)	158 (49.22)		
Female	326 (50.78)	163 (50.78)		
**Smoking, n (%)**			0.227	0.634
Yes	230 (35.83)	110 (34.27)		
No	412 (64.17)	211 (65.73)		
**Drinking, n (%)**			0.004	0.951
Yes	105 (16.36)	53 (16.51)		
No	537 (83.64)	268 (83.49)		
**Duration, months, median (IQR)**	–	48.00 (115.00)	–	–

BMI, body mass index; IQR, interquartile range. Data are presented as median ± IQR and n (%). Data are analyzed by Mann-Whitney *U* test and chi-square test.

Supplemental Table 2. Baseline characteristics of vitiligo patients with different activity

**Characteristics**	**Stable (n = 108)**	**Progressive (n = 213)**	***U*-Value/ χ^2-Value**	***p*-Value**
**Age, years, median (IQR)**	36.00 (13.00)	37.00 (15.00)	10708.000	0.312
**BMI, kg/m^2, median (IQR)**	22.79 (4.41)	23.94 (4.93)	10221.500	0.103
**Duration, months, median (IQR)**	60.00 (108.00)	48.00 (114.00)	9890.000	0.040[A]
**Gender, n (%)**			2.118	0.146
Male	47 (43.52)	111 (52.11)		
Female	61 (56.48)	102 (47.89)		
**Smoking, n (%)**			0.996	0.318
Yes	33 (30.56)	77 (36.15)		
No	75 (69.44)	136 (63.85)		
**Drinking, n (%)**			1.016	0.313
Yes	21 (19.44)	32 (15.02)		
No	87 (80.56)	181 (84.98)		

BMI, body mass index; IQR, interquartile range. Data are presented as median ± IQR and n (%). Data are analyzed by Mann-Whitney *U* test and chi-square test. [A]Statistically significant at $p < 0.05$.

Supplemental Table 3. Baseline characteristics of vitiligo patients with different severity

Characteristics	Mild (n = 119)	Moderate (n = 157)	Severe (n = 45)	*H*-Value/ *F*-Value/ χ^2-Value	*p*-Value
**Age, years, median (IQR)**	33.00 (13.00)	38.00 (13.50)	40.00 (13.00)	17.871	<0.001[A]
**BMI, kg/m^2, mean (SD)**	23.42 (4.75)	23.71 (4.54)	23.71 (5.04)	0.403	0.817
**Duration, months, median (IQR)**	24.00 (48.00)	60.00 (120.00)	180.00 (216.00)	47.946	<0.001[A]
**Gender, n (%)**				1.115	0.573
Male	55 (46.22)	82 (52.23)	21 (46.67)		
Female	64 (53.78)	75 (47.77)	24 (53.33)		
**Smoking, n (%)**				3.453	0.178
Yes	42 (35.29)	58 (36.94)	10 (22.22)		
No	77 (64.71)	99 (63.06)	35 (77.78)		
**Drinking, n (%)**				2.277	0.320
Yes	24 (20.17)	24 (15.29)	5 (11.11)		
No	95 (79.83)	133 (84.71)	40 (88.89)		

BMI, body mass index; IQR, interquartile range; SD, standard deviation. Data are presented as median ± IQR, mean ± SD, and n (%). Data are analyzed by Kruskal-Wallis *H* test, one-way ANOVA, and chi-square test. [A]Statistically significant at $p < 0.05$.

Supplemental Table 4. Baseline characteristics of vitiligo patients with different type

Characteristics	Segmental (n = 36)	Non-segmental (n = 277)	U-Value/ χ^2-Value	p-Value
**Age, years, median (IQR)**	33.50 (15.00)	37.00 (14.00)	4186.00	0.117
**BMI, kg/m^2, median (IQR)**	22.87 (4.33)	23.73 (4.50)	4230.50	0.139
**Duration, months, median (IQR)**	36.00 (57.00)	60.00 (120.00)	3847.50	0.026[A]
**Gender, n (%)**			3.777	0.052
Male	12 (33.33)	140 (50.54)		
Female	24 (66.67)	137 (49.46)		
**Smoking, n (%)**			0.199	0.656
Yes	11 (30.56)	95 (34.30)		
No	25 (69.44)	182 (65.70)		
**Drinking, n (%)**			0.015	0.904
Yes	6 (16.67)	44 (15.88)		
No	30 (83.33)	233 (84.12)		

BMI, body mass index; IQR, interquartile range. Data are presented as median ± IQR and n (%). Data are analyzed by Mann-Whitney U test and chi-square test. [A]Statistically significant at $p < 0.05$.

Supplemental Table 5. Incidence of hyperglycemia in vitiligo patients and healthy controls

Characteristic	Healthy (n = 642)	Vitiligo (n = 321)	χ^2-Value	OR	95%CI	p-Value
**FBG, n (%)**			6.335	1.781	1.131-2.806	0.012[A]
≤ 6.11 mmol/L	597 (92.99)	283 (88.16)				
> 6.11 mmol/L	45 (7.01)	38 (11.84)				

FBG, fasting blood glucose; OR, odds ratio; 95%CI, 95% confidence interval. Data are presented as n (%). Data are analyzed by chi-square test. [A]Statistically significant at $p < 0.05$.

Supplemental Table 6. Incidence of hyperglycemia in vitiligo patients with different activity

Characteristic	Stable (n = 108)	Progressive (n = 213)	χ^2-Value	OR	95%CI	*p*-Value
**FBG, n (%)**			6.155	3.006	1.216-7.430	0.013[A]
≤ 6.11 mmol/L	102 (94.44)	181 (84.98)				
> 6.11 mmol/L	6 (5.56)	32 (15.02)				

FBG, fasting blood glucose; OR, odds ratio; 95%CI, 95% confidence interval. Data are presented as n (%). Data are analyzed by chi-square test. [A]Statistically significant at $p < 0.05$.

Supplemental Table 7. Incidence of hyperglycemia in vitiligo patients with different severity

Characteristic	Mild (n = 119)	Moderate (n = 157)	Severe (n = 45)	Pearson's R	χ^2-Value	*p*-Value
**FBG, n (%)**				0.196	12.308	<0.001[A]
≤ 6.11 mmol/L	115 (96.64)	132 (84.08)	36 (80.00)			
> 6.11 mmol/L	4 (3.36)	25 (15.92)	9 (20.00)			

FBG, fasting blood glucose. Data are presented as n (%). Data are analyzed by chi-square test.

[A]Statistically significant at $p < 0.05$.

Supplemental Table 8. Incidence of hyperglycemia in vitiligo patients with different type

Characteristic	Segmental (n = 36)	Non-segmental (n = 277)	χ^2-Value	OR	95%CI	*p*-Value
**FBG, n (%)**			1.654	2.539	0.585-11.027	0.198
≤ 6.11 mmol/L	34 (94.44)	241 (87.00)				
> 6.11 mmol/L	2 (5.56)	36 (13.00)				

FBG, fasting blood glucose; OR, odds ratio; 95%CI, 95% confidence interval. Data are presented as n (%). Data are analyzed by chi-square test.

Supplemental Table 9. Baseline characteristics of the participants in the targeted liquid chromatography-mass spectrometry metabolomics analysis

Characteristics	Healthy (n = 30)	Vitiligo (n = 30)	*U*-Value/ χ^2-Value	*p*-Value
**Age, years, median (IQR)**	38.50 (17.80)	39.00 (15.30)	445.000	0.941
**BMI, kg/m^2, median IQR)**	23.94 (3.17)	22.92 (5.24)	431.500	0.784
**Gender, n (%)**			<0.001	1.000
Male	18 (60.00)	18 (60.00)		
Female	12 (40.00)	12 (40.00)		
**Smoking, n (%)**			0.739	0.390
Yes	10 (33.33)	7 (23.33)		
No	20 (66.67)	23 (76.67)		
**Drinking, n (%)**			3.354	0.067
Yes	10 (33.33)	4 (13.33)		
No	20 (66.66)	26 (86.67)		

BMI, body mass index; IQR, interquartile range. Data are presented as median ± IQR and n (%). Data are analyzed by Mann-Whitney *U* test and chi-square test.

Supplemental Figure 1

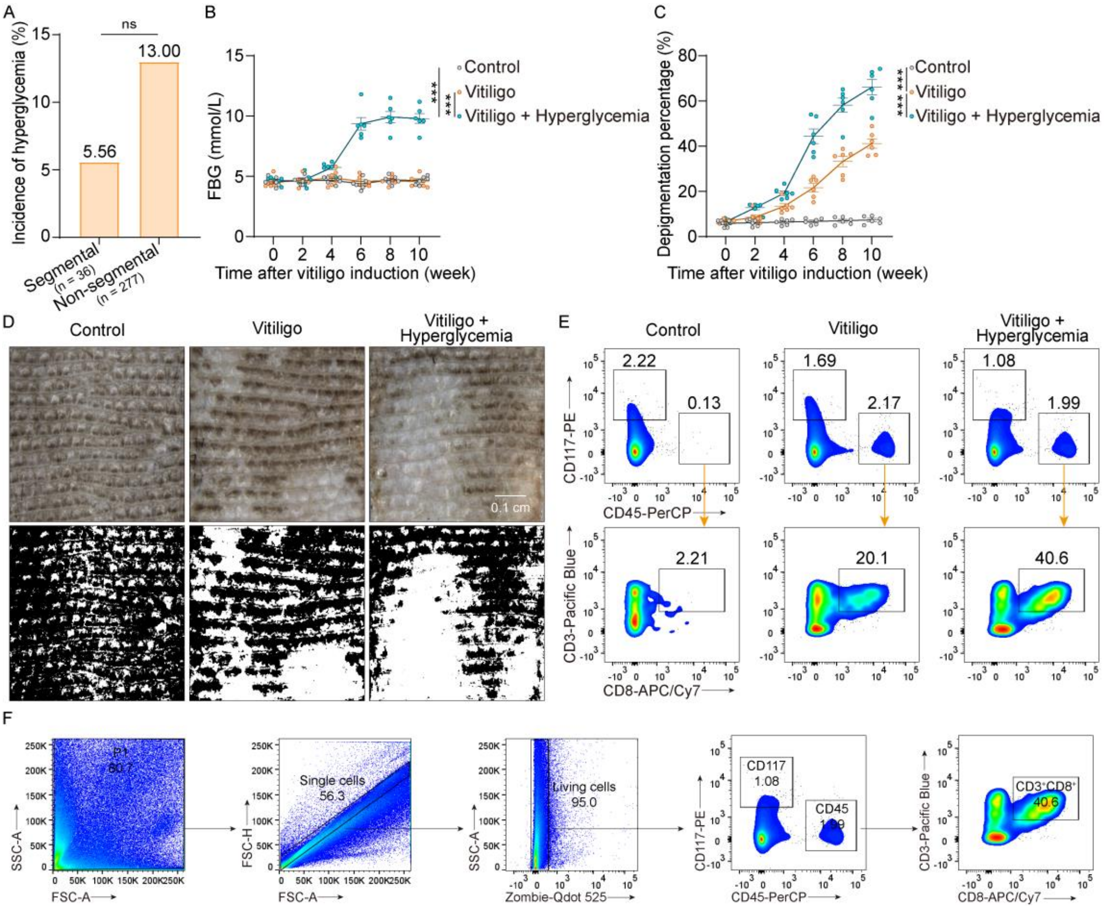

Supplemental Figure 1. Hyperglycemia promotes depigmentation of vitiligo. (**A**) Incidence of hyperglycemia in vitiligo patients with different types. (**B** and **C**) Longitudinal measurements of FBG levels (B) and tail depigmentation percentages (C) of mice from each group (n = 6) over 10 weeks. (**D**) Representative tail skin images of mice in each group at week 10. (**E**) Frequencies of $CD45^{-}CD117^{+}$ melanocytes and $CD3^{+}CD8^{+}$ T cells in tail epidermis of mice from each group. (**F**) Gating strategy to determine the frequencies of $CD45^{-}CD117^{+}$ and $CD3^{+}CD8^{+}$ T cells in tail epidermis of mice. Data shown are representative of the Vitiligo + Hyperglycemia group. Data are presented as frequency (A) or mean ± SEM (B and

C), and analyzed by chi-square test (A), Student's *t*-test (B), or one-way ANOVA (C).

$^{***}p < 0.001$; ns, not significant. FBG, fasting blood glucose.

Supplemental Figure 2

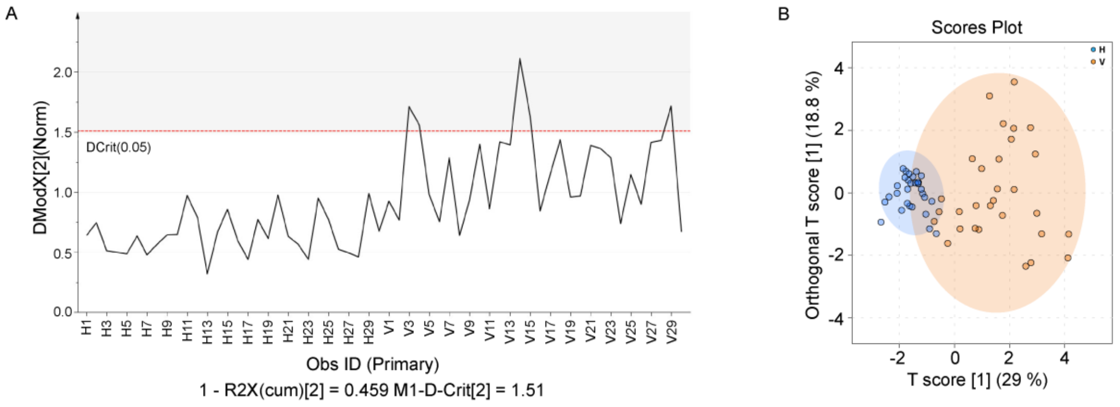

Supplemental Figure 2. Targeted metabolomic analyses on serum samples from vitiligo patients and healthy individuals. (**A**) DModX of each sample calculated by SIMCA software. (**B**) OPLS-DA score plot of serum metabolites from vitiligo patients and age- and sex-matched healthy controls (n = 30). Vitiligo patients are shown in orange and healthy controls are shown in blue. The model parameters are $R^2Y = 0.725$, $Q^2 = 0.659$.

Supplemental Figure 3

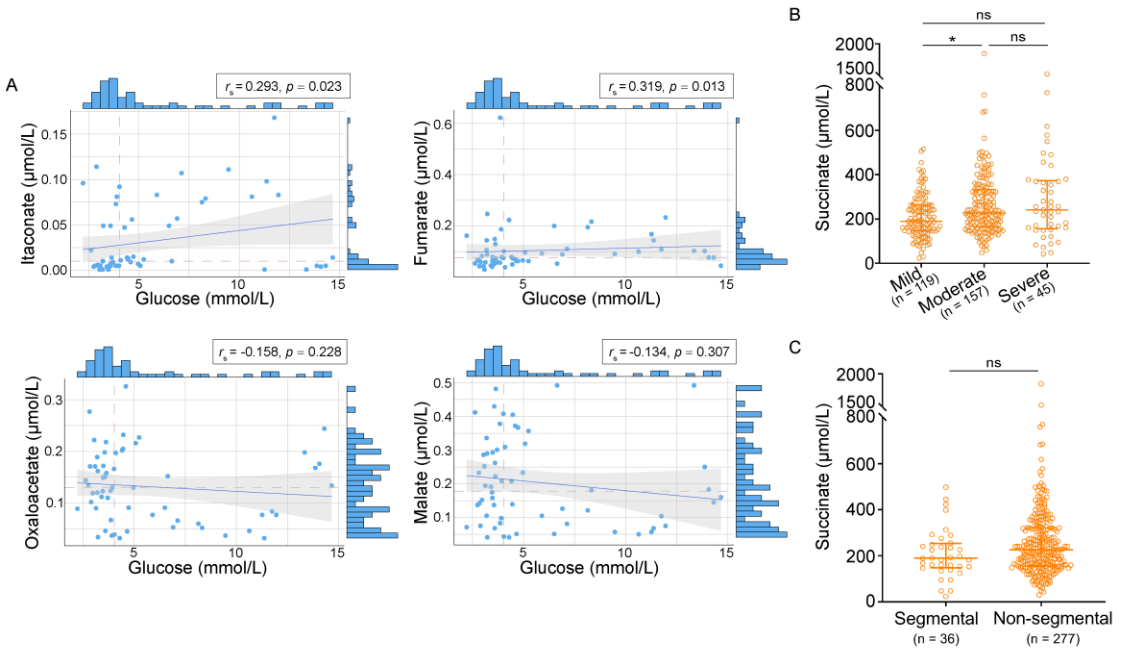

Supplemental Figure 3. Succinate is the key metabolite associated with hyperglycemia-aggravated vitiligo. (**A**) Correlation between serum glucose levels and concentrations of itaconate, fumarate, oxaloacetate, and malate in vitiligo patients and healthy controls (n = 60). (**B** and **C**) Serum succinate levels of vitiligo patients with different severity (B) and vitiligo patients with different types (C). Data are presented as median ± IQR and analyzed by Kruskal-Wallis *H* test (B and C). $^{*}p < 0.05$; ns, not significant. DModX, distance to model X; OPLS-DA, orthogonal partial least squares discriminant analysis.

Supplemental Figure 4

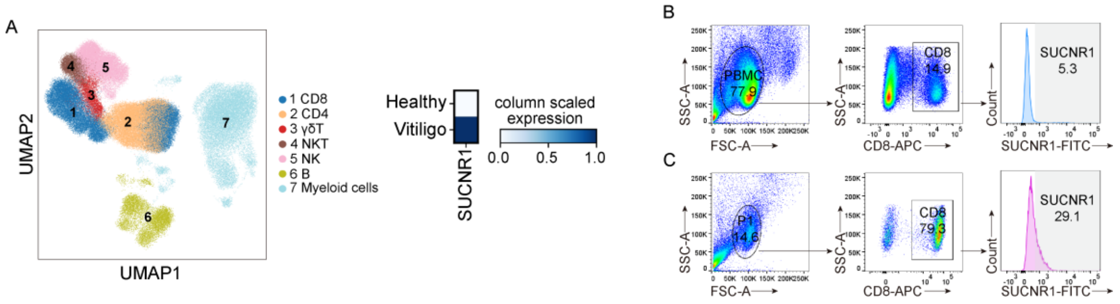

Supplemental Figure 4. Elevated SUCNR1 expression in CD8$^+$ T cells from vitiligo patients. (**A**) Cell type annotation and SUCNR1 expression in CD8$^+$ T cells from vitiligo patients and healthy controls, based on scRNA-seq data. (**B**) Gating strategy for determining the frequency of SUCNR1$^+$ cells among CD8$^+$ T cells in PBMCs from vitiligo patients and healthy controls. Data shown are representative of the healthy control group. (**C**) Gating strategy for determining the frequency of SUCNR1$^+$ cells among CD8$^+$ T cells from vitiligo patients treated with high glucose or succinate. Data shown are representative of the succinate-treated group. scRNA-seq, single-cell RNA-sequencing; PBMCs, peripheral blood mononuclear cells.

Supplemental Figure 5

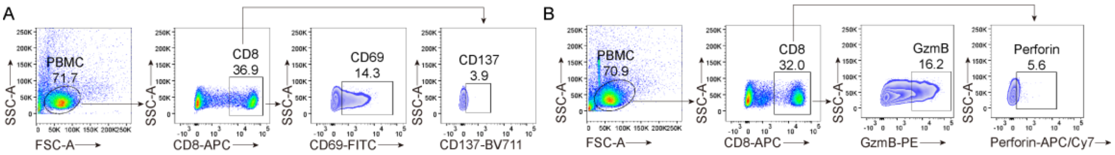

Supplemental Figure 5. Gating strategy for determining CD8⁺ T cell activation and effector function. (**A** and **B**) Gating strategy to determine the frequencies of CD69⁺ and CD137⁺ cells (A), as well as GzmB⁺ and Perforin⁺ cells (B), among CD8⁺ T cells. PBMCs were isolated from vitiligo patients pre-treated with NF-56-EJ40, followed by treatment with high glucose or succinate. Data shown are representative of the control group. GzmB, granzyme B; PBMCs, peripheral blood mononuclear cells.

Supplemental Figure 6

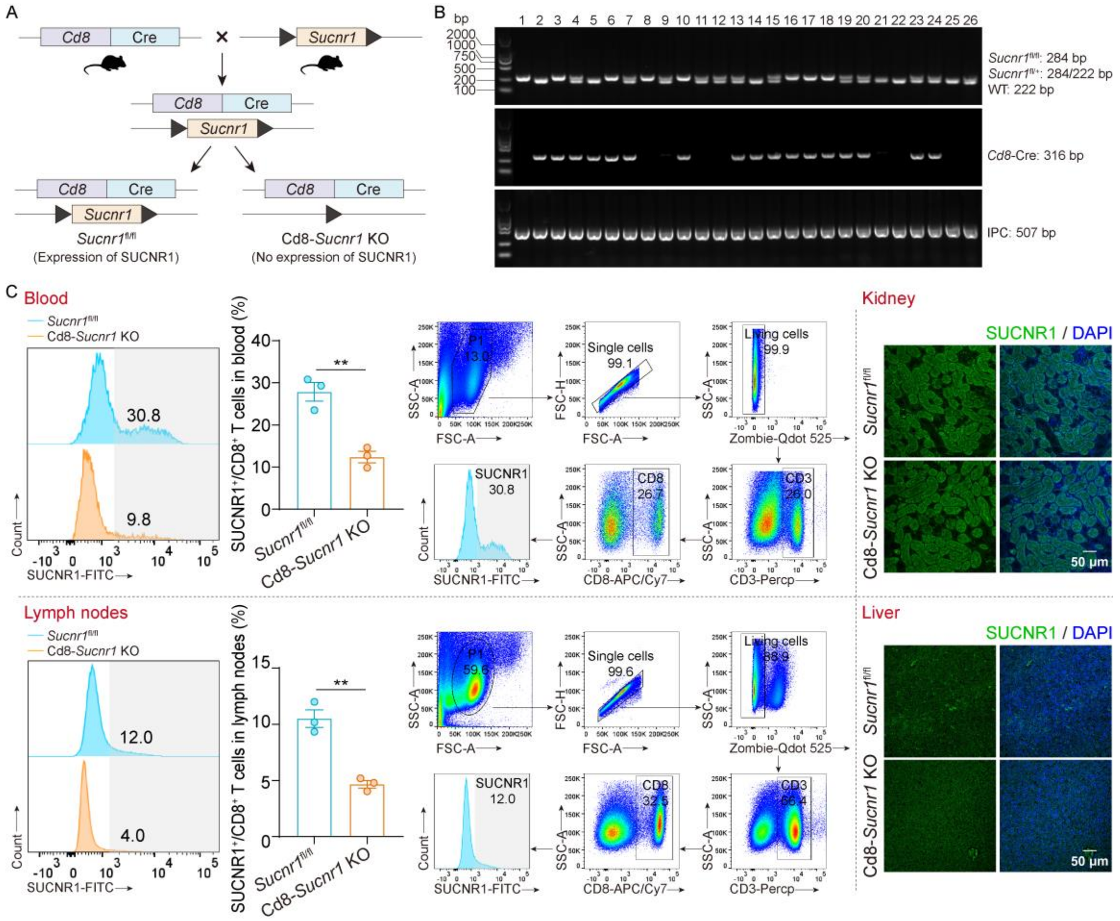

Supplemental Figure 6. Generation of Cd8-specific deletion of *Sucnr1* in mice.

(**A**) Generation of *Sucnr1*$^{fl/fl}$ and Cd8-*Sucnr1* KO mice. (**B**) Genotyping of *Sucnr1*$^{fl/fl}$ and Cd8-*Sucnr1* KO mice (Flox allele = 284 bp; Cre allele = 316 bp; IPC allele = 507 bp). (**C**) SUCNR1 expression in blood and lymph nodes in Cd8-*Sucnr1* KO mice and age-matched *Sucnr1*$^{fl/fl}$ littermates. The expression of SUCNR1 (green) in kidney and liver in Cd8-*Sucnr1* KO mice and age-matched *Sucnr1*$^{fl/fl}$ littermates. Data are presented as mean ± SEM and analyzed by Student's *t*-test (C). $^{**}p < 0.01$. IPC, internal positive control.

Supplemental Figure 7

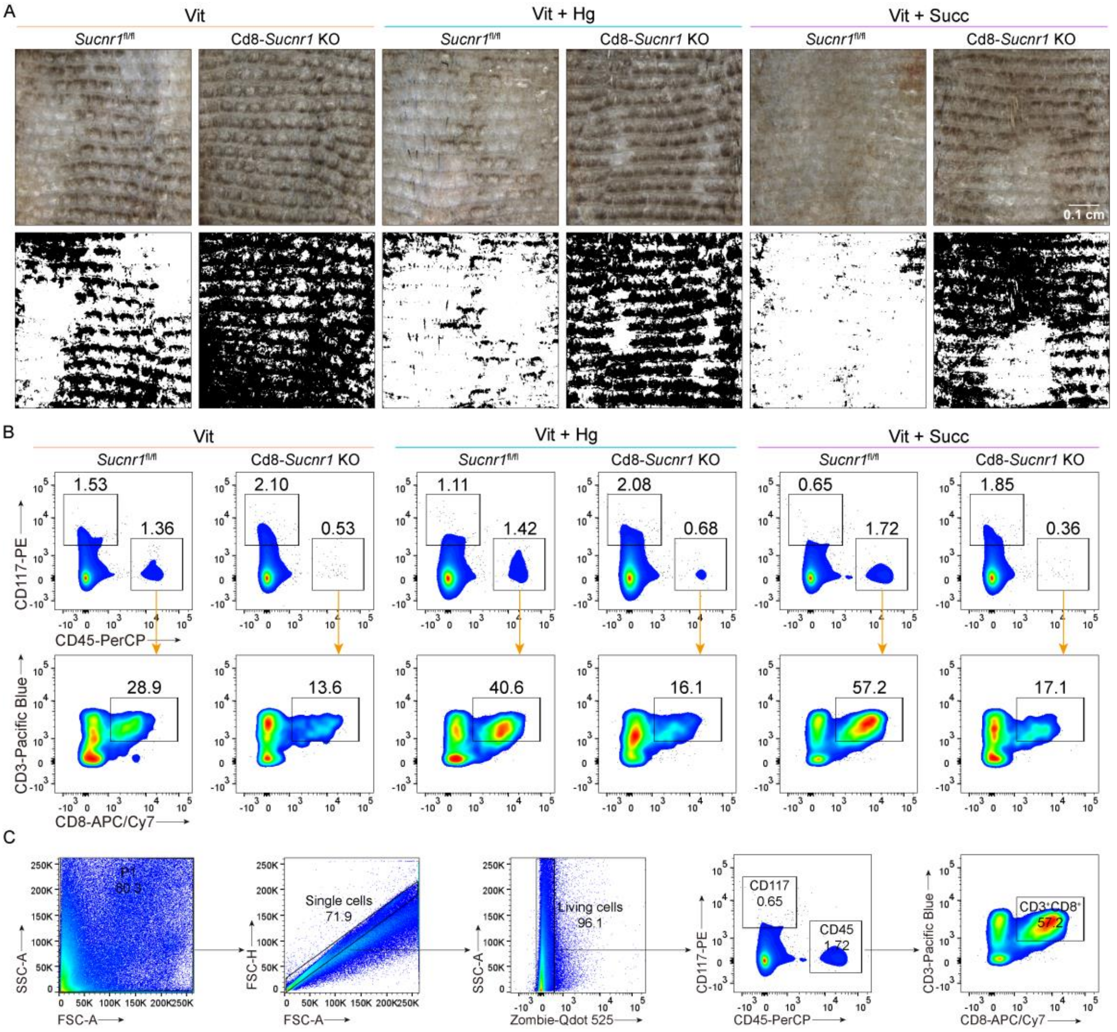

Supplemental Figure 7. Succinate promotes vitiligo depigmentation and CD8$^+$ T cell infiltration through SUCNR1. (**A**) Representative tail skin images of mice in each group at week 10. (**B**) Frequencies of CD45$^-$CD117$^+$ melanocytes and CD3$^+$CD8$^+$ T cells in tail epidermis of mice from each group. (**C**) Gating strategy to determine the frequencies of CD45$^-$CD117$^+$ melanocytes and CD3$^+$CD8$^+$ T cells in tail epidermis of mice. Data shown are representative of the *Sucnr1*$^{fl/fl}$-Vit + Succ group. Vit, vitiligo; Succ, succinate.

Supplemental Figure 8

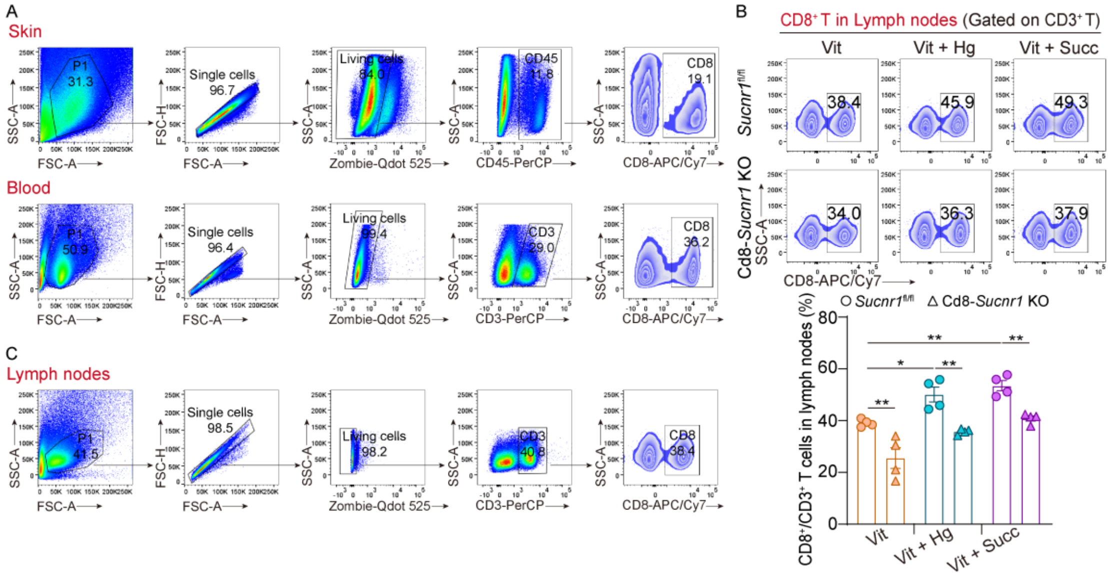

Supplemental Figure 8. Gating strategy for CD8$^+$ T cells in tissues. (**A**) Gating strategy to determine the frequency of CD8$^+$ T cells in tail skin and blood of mice. Data shown are representative of the *Sucnr1*$^{fl/fl}$-Vit + Hg group. (**B**) Frequency of CD8$^+$ T cells in lymph nodes of mice from each group (n = 4). (**C**) Gating strategy to determine the frequency of CD8$^+$ T cells in lymph nodes of mice. Data shown are representative of the *Sucnr1*$^{fl/fl}$-Vit group. Data are presented as mean ± SEM and analyzed by one-way ANOVA (B). $^*p < 0.05$, $^{**}p < 0.01$. Vit, vitiligo; Hg, hyperglycemia.

Supplemental Figure 9

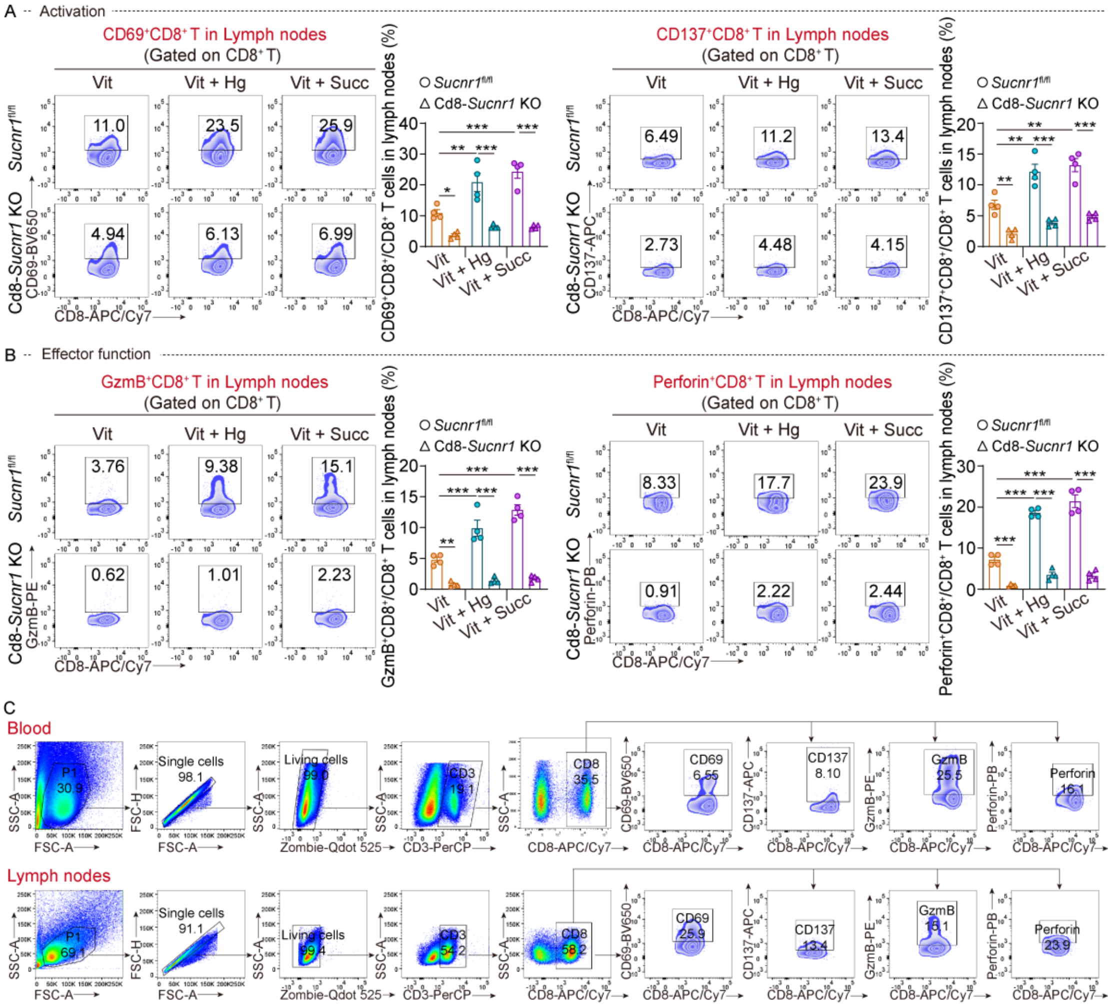

Supplemental Figure 9. Succinate induces CD8$^+$ T cell hyperactivation in blood and lymph nodes through SUCNR1. (**A** and **B**) Flow cytometry analysis of CD69$^+$CD8$^+$ T cells, CD137$^+$CD8$^+$ T cells (A), GzmB$^+$CD8$^+$ T cells, and Perforin$^+$CD8$^+$ T cells (B) in lymph nodes of mice from each group (n = 4). (**C**) Gating strategy for determining the frequencies of CD69$^+$, CD137$^+$, GzmB$^+$, and Perforin$^+$ cells among CD8$^+$ T cells in blood and lymph nodes. Data shown are representative of the *Sucnr1*$^{fl/fl}$-Vit + Hg and *Sucnr1*$^{fl/fl}$-Vit + Succ groups, respectively. Data are presented as mean ± SEM and analyzed by one-way ANOVA

(A and B). $^{*}p < 0.05$, $^{**}p < 0.01$, $^{***}p < 0.001$. GzmB, granzyme B; Vit, vitiligo; Hg, hyperglycemia; Succ, succinate.

Supplemental Figure 10

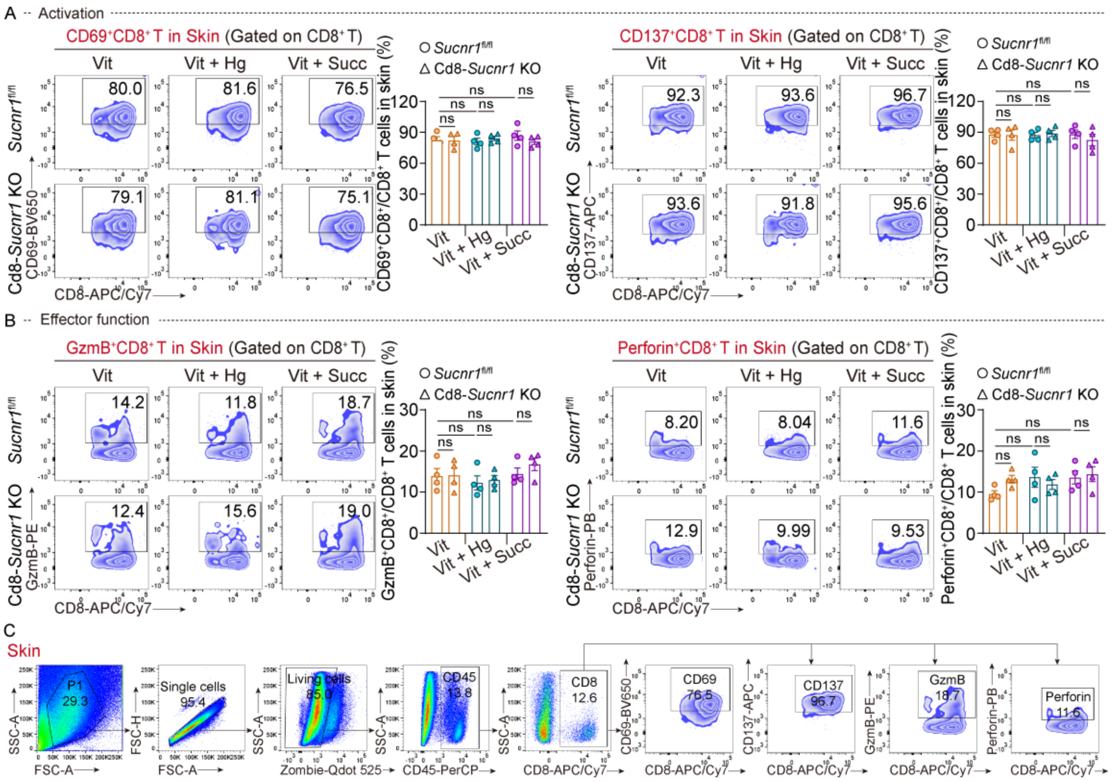

Supplemental Figure 10. The binding of succinate to SUCNR1 has no significant effect on the activation and effector function of CD8⁺ T cells in tail skin. (**A** and **B**) Flow cytometry analysis of CD69$^+$CD8$^+$ T cells, CD137$^+$CD8$^+$ T cells (A), GzmB$^+$CD8$^+$ T cells, and Perforin$^+$CD8$^+$ T cells (B) in tail skin of mice from each group (n = 4). (**C**) Gating strategy for determining the frequencies of CD69$^+$, CD137$^+$, GzmB$^+$, and Perforin$^+$ cells among CD8$^+$ T cells in tail skin. Data shown are representative of the *Sucnr1*$^{fl/fl}$-Vit + Succ group. Data are presented as mean ± SEM and analyzed by one-way ANOVA (A and B). ns, not significant. GzmB, granzyme B; Vit, vitiligo; Succ, succinate.

Supplemental Figure 11

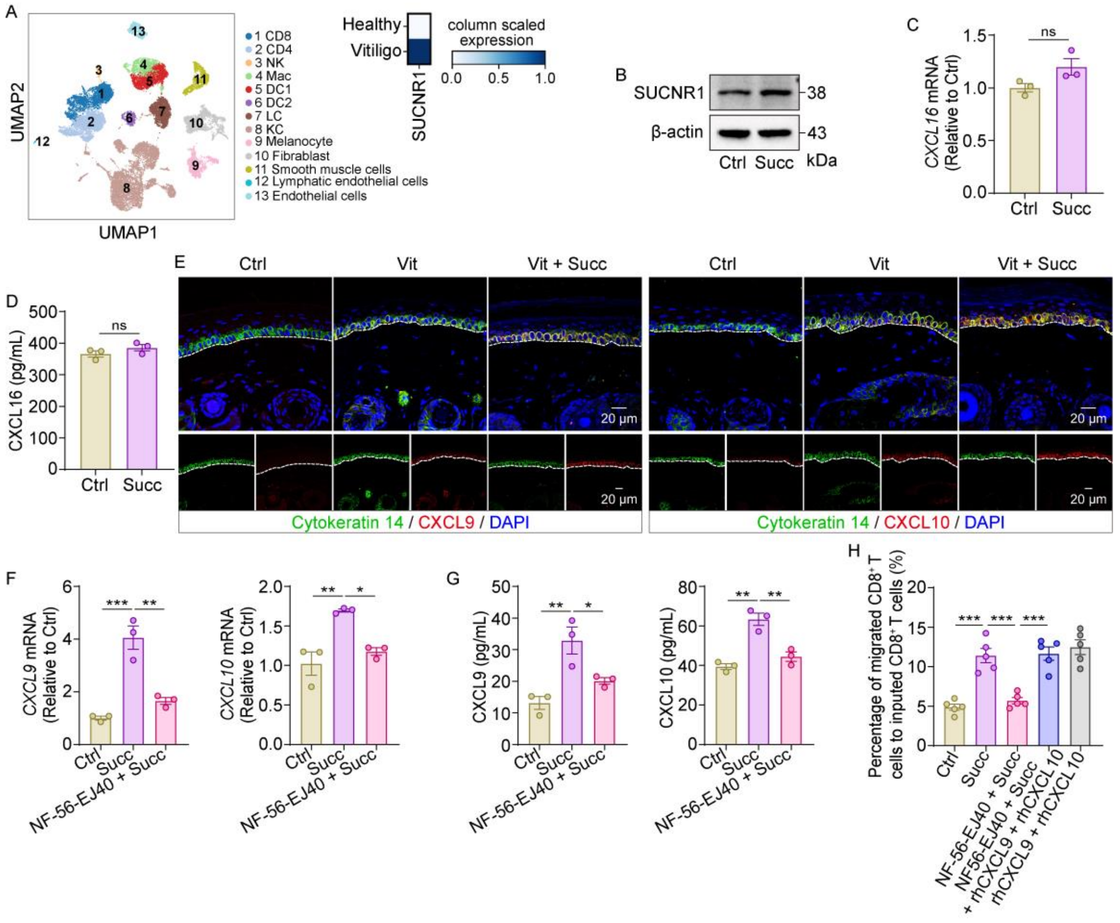

Supplemental Figure 11. Succinate-induced chemokine secretion via SUCNR1 in keratinocytes promotes CD8$^+$ T cells migration. (**A**) Cell type annotation of scRNA-seq data and the expression of SUCNR1 in keratinocytes in vitiligo patients and healthy controls. (**B**) The protein level of SUCNR1 in NHKs treated with succinate. (**C** and **D**) The mRNA (C) and the secretion (D) levels of CXCL16 in NHKs treated with succinate. (**E**) The expression of CXCL9 and CXCL10 (red) in keratinocytes of mouse tail skin from each group. Keratinocytes were characterized by cytokeratin 14 (green), nuclei were counterstained with DAPI (blue). (**F** and **G**) The mRNA (F) and secretion (G) levels of CXCL9 and CXCL10 in NHKs treated

with succinate or pretreated with SUCNR1 inhibitor NF-56-EJ40 prior to succinate stimulation. (**H**) Transwell assay showing the percentage of migrated $CD8^+$ T cells in response to culture supernatants from succinate-treated NHKs with NF-56-EJ40 or with the addition of rhCXCL9 or rhCXCL10 into the transwell system. Data are presented as mean ± SEM (C, D, and F-H) and analyzed by Student's *t*-test (C and D) or one-way ANOVA (F-H). $^{*}p < 0.05$; $^{**}p < 0.01$; $^{***}p < 0.001$; ns, not significant. scRNA-seq, single-cell RNA-sequencing; NHKs, normal human keratinocytes; Ctrl, control; Succ, succinate; rhCXCL9, recombinant human CXCL9; rhCXCL10, recombinant human CXCL10.

Supplemental Figure 12

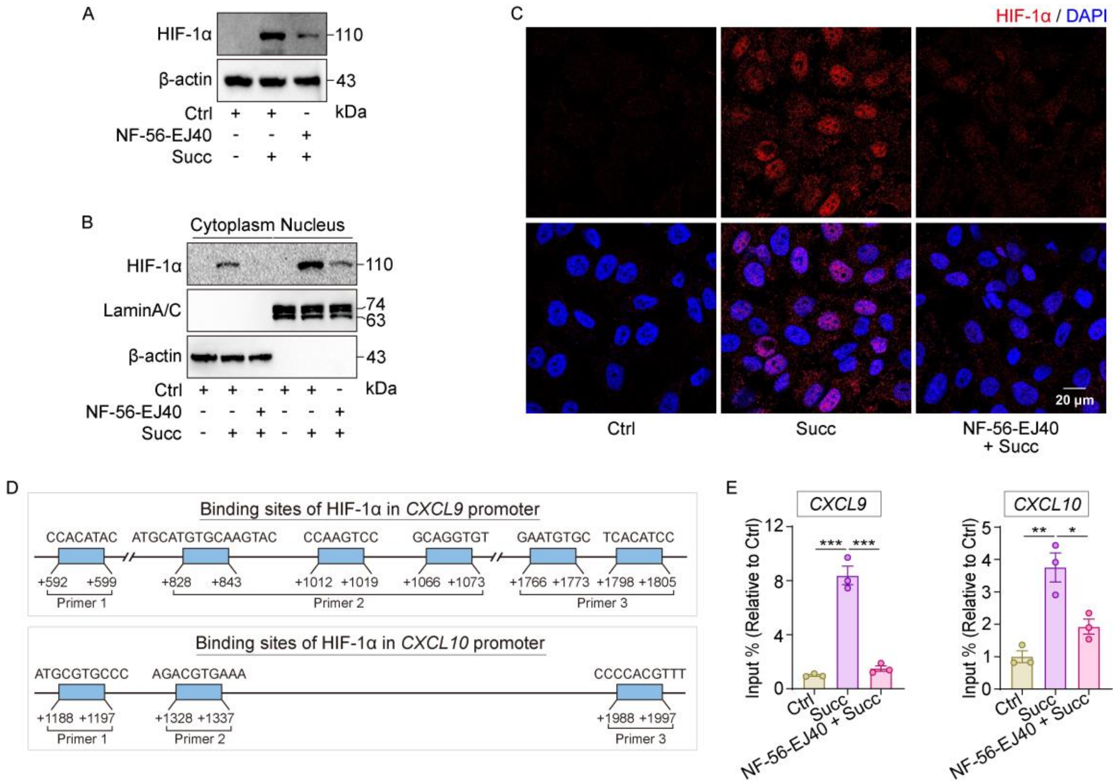

Supplemental Figure 12. Succinate promotes HIF-1α nuclear translocation and subsequent binding to the CXCL9 and CXCL10 promoter regions through SUCNR1. (**A**) Expression of HIF-1α in NHKs pretreated with NF-56-EJ40 prior to succinate stimulation. (**B**) Expression of HIF-1α in the cytoplasm and nucleus of NHKs pretreated with NF-56-EJ40 prior to succinate stimulation. (**C**) Expression of HIF-1α in NHKs pretreated with NF-56-EJ40 before succinate stimulation. (**D**) Binding sites of HIF-1α in *CXCL9* and *CXCL10* promoter region. (**E**) Binding of HIF-1α to *CXCL9* and *CXCL10* promoter region in NHKs pretreated with NF-56-EJ40 prior to succinate treatment. Data are presented as mean ± SEM and analyzed by one-way ANOVA (E). $^{*}p < 0.05$, $^{**}p < 0.01$, $^{***}p < 0.001$. NHKs, normal human keratinocytes; Ctrl, control; Succ, succinate.

Supplemental Methods

Patients and clinical samples. The hospital-based case-control study included 321 newly diagnosed vitiligo patients in the Department of Dermatology of Xijing Hospital and 642 healthy volunteers matched on age (± 2 years) and sex in the Physical Examination Center of Xijing Hospital of the Fourth Military Medical University from 2020 to 2023. Individuals receiving systemic corticosteroids or any systemic treatment in the past three months, as well as patients with active autoimmune diseases, and a history of any chronic illness, including neoplastic, collagen, and hematologic disorders, and pregnant and lactating females were excluded. Epidemiological data, including age, gender, vitiligo duration, Vitiligo Disease Activity (VIDA) score, Body Surface Area (BSA), Vitiligo Area Scoring Index (VASI), vitiligo types and treatments were collected through a questionnaire survey. The VIDA score, BSA, and VASI were evaluated by two senior dermatologists and calculated based on the average. According to the worldwide expert recommendations for vitiligo diagnosis and management, disease activity was defined by the VIDA score, and its severity was defined by the percentage of BSA involvement (59). Serum samples were collected from all individuals. Blister fluid was obtained from vitiligo patients who underwent autologous transplantation of non-cultured epidermal cell suspension. For each vitiligo patient, we collected two sets of blister fluid, one lesional sample from affected skin and one non-lesional sample from unaffected skin, located at least 10 cm away from any visible lesion.

Generation of Cd8-specific deletion of Sucnr1 in mice. Cd8 cell-specific Sucnr1 knockout mice (Cd8-*Sucnr1* KO) were generated by breeding Cd8-Cre mice (C001333, Cyagen) with *Sucnr1*$^{fl/fl}$ mice (CKOCMP-84112-Sucnr1-B6J-VA, Cyagen). The mouse genotypes were identified by PCR amplification of DNA extracted from toes. Primer sequences are listed as follows: Cd8-Cre (forward 5'-GCTGGAGTTTCAATACCGGAGATC-3' and reverse: 5'-GGAAACGCAACGGATATTGAGTC-3'), Internal Positive Control (forward 5'-CTATCAGGGATACTCCTCTTTGCC-3' and reverse: 5'-GATACAGGAATGACAAGCTCATGGT-3'), Sucnr1 Loxp-1 (forward 5'-TGACTCCTCAACACTAAATCTGGA-3' and reverse: 5'-GTAACCAAATCTTGTGCTCACCG-3').

Induction of vitiligo mice and treatment. The vitiligo mice model was established as previously described (32–34). Briefly, wild-type (purchased from the Animal Center of the Fourth Military Medical University) or gene-knockout C57BL/6J mice (female, 8-10 weeks old, 18-20 g) were intradermally injected with 2×10^5 B16F10 cells (gifted by Dr. Qianjin Lu, Institute of Dermatology, Chinese Academy of Medical Sciences and Peking Union Medical College, Nanjing, China) (cell viability was more than 96 % characterized by harvesting after limited passage in vitro and off-white to white color of cell mass after centrifugation) into the left-back on day 0.

Then, mice were intraperitoneally injected with anti-mouse CD4 antibody (Clone GK1.5, BioXCell) at 10 μg/g to eliminate regulatory T cells on day 4 and day 10. Subsequently, tumors were surgically excised on day 12. For hyperglycemia induction, after tumor resection, 100 mg/kg streptozotocin (STZ) (S0130, Sigma-Aldrich) was administered via an intraperitoneal injection after a high-fat diet (HF60, Dyets) for 4 weeks. Three days after STZ injection, mice with fasting blood glucose ≥ 5.0 mmol/L and ≤ 11.1 mmol/L were considered hyperglycemic. The succinate treatment group received 100 mg/kg succinate (S8250, Solarbio) intraperitoneal injection twice weekly for 10 weeks after tumor excision. The extent of depigmentation was objectively quantified with the percentage of the anatomic site (tail) using Image J software (images converted to 8-bit black on pigmented areas and white on depigmented areas). All mice were housed in micro isolator cages in a specific pathogen-free setting at 24 ± 2 ˚C and exposed to a 12 h light/12 h dark cycle, with standard feed and water provided ad libitum at the Experimental Animal Center of Fourth Military Medical University.

Cell culture and treatments. Normal human keratinocytes (NHKs) were isolated from the foreskins donated by healthy individuals who elected for circumcision in the Department of Urology of Xijing Hospital. All the donors provided informed consent. NHKs were cultured in basal culture medium for the growth of normal human keratinocytes (2277436, Gibco) with 1 % growth supplement (2496262, Gibco).

Second to fifth passage NHKs were used, and each experiment was repeatedly performed with at least three different donors. Cells were cultured in a humidified atmosphere with 5 % CO_2 at 37 °C. NHKs were treated with 5 mM succinate (S8250, Solarbio) or pretreated with 50 nM NF-56-EJ40 (HY-130246, MedChemExpress), the human-specific SUCNR1 antagonist, for 3 h prior to succinate treatment. Transfection of small interfering RNA (siRNA) for SUCNR1 (sc-62407, Santa Cruz Biotechnology) or HIF-1α (sc-35561, Santa Cruz Biotechnology) and plasmids for HIF-1α (6*His, NM_001530.4, Tsingke) were transfected into cells with lipofectamine 3000 (L3000015, Invitrogen).

Peripheral Blood Mononuclear Cells (PBMCs) and CD8$^+$ T cells isolation and treatment. PBMCs were isolated from the peripheral blood of vitiligo patients and healthy controls using Ficoll density gradient centrifugation with lymphocyte separation solution (7111011, Dakewe) according to the manufacturer's instructions. CD8$^+$ T cells were isolated from PBMCs using the Human CD8$^+$ T Cell Isolation Kit (130-096-495, Miltenyi Biotec) according to the manufacturer's instructions. PBMCs or CD8$^+$ T cells were cultured in RPMI-1640 (SH30809.01, Hyclone) supplemented with 10 % fetal bovine serum (FBS) (16140089, Gibco) and 0.1 % penicillin-streptomycin. Cells were treated with high glucose medium containing 4 g/L glucose, 5 mM succinate (S8250, Solarbio), or pretreated with 50 nM NF-56-EJ40 (HY-130246, MedChemExpress) for 3 h prior to succinate treatment. Glucose (G8150,

Solarbio) was added to RPMI 1640 (originally 2 g/L glucose) and filter sterilized to obtain a high glucose medium (4 g/L glucose). For the evaluation of activation and effect function, CD8$^+$ T cells were stimulated with ActSep® CD3/CD28 activation magnetic beads (GMP-TL601, Biotechnology).

Single-cell suspensions of mouse tail epidermis, mouse tail skin, peripheral blood, and lymph nodes. For single-cell suspension of mouse tail epidermis. The mouse tail skin was cut into 0.5 × 0.5-cm pieces, and then floated dermis-side down in 2 mg/mL Dispase II solution (04942078001, Roche) and incubated at 37 °C for 30 min. Then, transfer the skin to a petri dish with 10 mL of cold HBSS (14175095, Gibco) and carefully separate the epidermis from the dermis with a fine-tipped tweezer. The removed epidermis was floated dermis-side down in 5 mL 0.05 % Trypsin-EDTA solution (diluted with HBSS from 0.25 % Trypsin-EDTA) (T1300, Solarbio) at 37 °C for 10 min, and afterwards 5 mL HBSS containing 5 % FBS was added to neutralize Trypsin. Transfer the neutralizing solution containing epidermal fragments onto a 70-µm cell strainer (352350, Coring) and ground with a 2 mL syringe base. Rinsed with PBS buffer containing 1 % FBS and filtered into a 50 mL centrifuge tube. After centrifugation and resuspended to obtain the single-cell suspension.

For single-cell suspension of mouse tail skin. The mouse tail skin was cut into small pieces of approximately 1~2 mm after stripping the tailbone. Prepare enzyme

mix by adding 4.6 mL of serum-free RPMI 1640 (SH30809.01, Hyclone), 300 μL of Enzyme D, 50 μL of Enzyme R, and 25 μL of Enzyme A of the Multi Tissue Dissociation Kit (130-110-201, Miltenyi Biotec) into a gentleMACS™ C-Tube (130-093-237, Miltenyi Biotec). Then added the skin pieces into the C-Tube and ran the program 37C_cus_m_wskin. The digested skin was filtered through a 70-μm cell strainer (352350, Coring) and then centrifuged cell suspension at 300×g for 10 min. Aspirated the supernatant completely and resuspended the cells in phosphate-buffered saline (PBS) buffer containing 1 % FBS for further analysis.

For single-cell suspension of mouse peripheral blood. Peripheral blood was collected by extracting the eyeball. Red blood cells were removed by using ammonium chloride potassium (ACK) lysing buffer. After washing with PBS and centrifugation, the pellet was resuspended to obtain a single-cell suspension.

For single-cell suspension of mouse lymph nodes. The skin was longitudinally cut and the inguinal lymph nodes were stripped. Placed the lymph nodes on the 70-μm cell strainer (352350, Coring) and ground with a 2 mL syringe base. Rinsed with PBS buffer containing 1 % FBS and filtered into a 50 mL centrifuge tube. After centrifugation and resuspended to obtain the single-cell suspension.

Fasting blood glucose (FBG) measurement. Serum samples of the case-control study subjects were collected after an 8-hour fasting period. The FBG levels were measured in the Department of Laboratory of Xijing Hospital. The FBG level higher

than 6.11 mmol/L was defined as hyperglycemia. After 16 h of fasting, blood samples were collected from the mouse tail tip, and the FBG levels were measured using a glucometer (Accu-Chek, Roche). The FBG level with a consistent range between 5.5 to 11.1 mmol/L is considered hyperglycemic.

Succinate colorimetric assay. Succinate levels in serum and blister fluid were detected by using a succinate assay kit (K-SUCC, Megazyme) according to the manufacturer's instructions. Briefly, succinate standard and samples were added to a 96-well plate. Then, regents 1~4 were added to per well in sequence. The absorbance was detected at 340 nm using a Model 680 Microplate Reader (Bio-Rad) and recorded as A1. Added regent 5 to each well, mixed gently, and let stand for 6 min. Absorbance at 340 nm was then followed every 5 min until the reaction was stable and recorded as A2. Calculate the succinate concentration according to the formula: Succinate concentration (μmol/L) = [(Sample A2 - Sample A1) - (Blank A2 - Blank A1)] / [(Standard A2 - Standard A1) - (Blank A2 - Blank A1)] * 0.2 / 118.09 * 10^6, where 118.09 is the molecular weight of succinate.

Whole-mount immunostaining and imaging of mouse tail epidermis. The mouse tail was cut off with a length of 1 cm, and the skin was flattened in a 12-well plate after removing hair with Nair™ hair removal cream and stripping the tailbone. Then, the skin was incubated in a 20 mM ethylenediaminetetraacetic acid (EDTA) solution

at 37 °C with shaking for 90 min. Then, carefully separate the epidermis from the dermis with a fine-tipped tweezer in the anterior-posterior direction and remove the hair follicles and sebaceous gland under the stereomicroscope. The isolated epidermis was then fixed in precooled 4 % paraformaldehyde for 10 min, washed with PBS three times (15 min per time), permeabilized with 0.3 % H_2O_2/methanol for 20 min at -20 °C whereafter washed with PBS as above. Afterward, block the skin in a solution of 2 % normal donkey serum, 1 % BSA, and 0.3 % Triton X-100 in PBS for 1 h with shaking at room temperature. This was followed by incubation with rabbit monoclonal antibody to Melan-A (1:300, ab210546, Abcam) and rat monoclonal antibody to CD8α (1:300, ab22378, Abcam) in the blocking buffer overnight at 4 °C. After washed with PBS, Cy3-Donkey Anti-Rabbit IgG (1:400, 711-165-152, Jackson ImmunoResearch Laboratories), Alexa Fluor 488-Donkey Anti-Rat IgG (1:400, 712-545-150, Jackson ImmunoResearch Laboratories) and 4', 6-diamidino-2-phenylindole (DAPI) (1:1000, C1006, Beyotime Biotechnology) in blocking solution were added for 8 h at 4 °C. Finally, the samples were mounted onto slides using glycerin and secured with cover glass, followed by washing with PBS. Images were captured in a sequential manner using the sequential scan mode using a confocal microscope (LSM 880, Carl Zeiss Microscopy), setting Z-stack to ensure that the z-position covers the entire thickness of samples. Data was analyzed using Imaris software and R Studio.

Quantitative real-time polymerase chain reaction (qRT-PCR) assay. Total RNA was extracted with Trizol reagent (15596018, Invitrogen). mRNA was reversely transcribed to cDNA with PrimeScript™ RT Master Mix kit (AK4301, TaKaRa). The qRT-PCR assay was performed using SYBR Premix Ex Taq™ II kit (AKA1008, TaKaRa) by the real-time PCR detection system (iQTM5, Bio-Rad). Relative gene expression was normalized to *ACTB* and calculated via the $2^{\wedge}(-\Delta\Delta Ct)$ method. Primer sequences are listed as follows: human *SUCNR1* (forward 5'-GCACTCAGGTCGTCATCAACTCC-3' and reverse: 5'-CAGCATGTCCCTGAAGTGATCTCC-3'), human *CXCL9* (forward 5'-TCTAAACCCAGATTCAGCA-3' and reverse: 5'-CTCCTTTGGAATGATAGCG-3'), human *CXCL10* (forward 5'-CCTCCAGTCTCAGCACCAT-3' and reverse: 5'-AAATTGGCTTGCAGGAATA-3'), human *CXCL16* (forward 5'-GCCATCGGTTCAGTTCA-3' and reverse: 5'-CAATCCCCGAGTAAGCAT-3'), human *HIF-1α* (forward 5'-AGTTCCGCAAGCCCTGAAAGC-3' and reverse: 5'-GCAGTGGTAGTGGTGGCATTAGC-3'), human *ACTB* (forward 5'-GGCTACAGCTTCACCACCAC-3' and reverse 5'-TGCGCTCAGGAGGAGC-3').

Western blot assay. After the indicated treatment, cells were washed three times with PBS and then lysed in RIPA lysis buffer (P0013C, Beyotime Biotechnology) containing 1 % phenylmethanesulfonyl fluoride (PMSF) (ST506, Beyotime Biotechnology) at 4 °C for 20 min. The supernatant was collected by centrifugation to

extract total cellular protein. Nuclear and cytosolic proteins were extracted using a nuclear and cytosolic protein extraction kit (P0027, Beyotime Biotechnology) according to the manufacturer's instructions. Protein concentrations were detected using the bicinchoninic acid protein assay kit (23227, Thermo Fisher Scientific). Equal amounts of protein were separated by sodium dodecyl sulfate-polyacrylamide gel electrophoresis (Bio-Rad) and transferred to polyvinylidene fluoride membranes (Millipore). After blocking with 5 % non-fat milk diluted in Tris-buffered saline with Tween-20 (TBST) (T1085, Solarbio) for 1 h at room temperature, the membranes were incubated with primary antibodies (1:1000) against SUCNR1 (NBP1-00861, Novus Biologicals), HIF-1α (D1S7W) (36169, Cell Signaling Technology), Hydroxy-HIF-1α (Pro564) (D43B5) (3434, Cell Signaling Technology), LaminA/C (10298-1-AP, Proteintech), and β-actin (8H10D10) (3700, Cell Signaling Technology) overnight at 4 °C. After washed with TBST three times, the membranes were incubated with corresponding secondary antibodies (1:5000) (Goat Anti-Rabbit IgG Antibody, Peroxidase Conjugated, AP132P, Sigma-Aldrich; Goat Anti-Mouse IgG Antibody, Peroxidase Conjugated, AP124P, Sigma-Aldrich) at room temperature for 1 h. The protein bands were detected and analyzed with the Western Blotting Imaging System (871BRO7308, Bio-Rad).

Flow cytometry. PBMCs or $CD8^+$ T cells were harvested after the indicated treatment, and single-cell suspensions were obtained from mouse tail epidermis,

mouse tail skin, peripheral blood, and lymph nodes. After washing with PBS, cells were incubated with fluorescent antibodies of surface molecules: APC anti-human CD8 antibody (344722, Biolegend), FITC anti-human/mouse SUCNR1 antibody (ASR-090-F-50UL, ThermoFisher Scientific), FITC anti-human CD69 antibody (310904, Biolegend), Brilliant Violet 711™ anti-human CD137 antibody (309832, Biolegend), Zombie Aqua™ Fixable Viability Kit (423101, Biolegend), PE anti-mouse CD117 antibody (161504, Biolegend), PerCP anti-mouse CD45 antibody (103130, Biolegend), Pacific Blue™ anti-mouse CD3 antibody (100214, Biolegend), PerCP anti-mouse CD3 antibody (100326, Biolegend), APC/Cyanine7 anti-mouse CD8 antibody (100714, Biolegend), Brilliant Violet 650™ anti-mouse CD69 antibody (104541, Biolegend), or APC anti-mouse CD137 antibody (106110, Biolegend) for 30 min. Followed with washing, cells were fixed with 100 μL Diluent Fixation/Permeabilization Concentrate (00-5523-00-2511819, Invitrogen) for 30 min. Following repeated washes, 1 mL Permeabilization Buffer (00-5523-00-2511274, Invitrogen) was added to incubate for 30 min. Subsequently, cells were incubated with fluorescent antibodies of intracellular molecules: PE anti-human/mouse Granzyme B (372208, Biolegend), APC/Cyanine7 anti-human Perforin antibody (308128, Biolegend), and Pacific Blue™ anti-mouse Perforin antibody (154312, Biolegend) for 30 min. All reactions were performed at room temperature and protected from light. Finally, the stained cells were washed and then resuspended in 400 μL PBS for flow cytometric analysis by BD LSRFortessa flow cytometer (BD Biosciences). Data were analyzed with FlowJo software.

Immunofluorescence assay. Paraffin-embedded sections (thickness, 5-μm) of skin samples were deparaffinized and antigens retrieved, followed by blocking with 5 % normal goat serum (AR0009, Boster) for 1 h at room temperature. For immunofluorescence staining of cultured cells, cells on coverslips were fixed in 4 % paraformaldehyde for 10 mins, permeabilized with 0.5 % Triton X-100 for 10 mins, and blocked with normal goat serum for 30 min. Subsequently, the sections or cells were incubated with primary antibodies (1:200) against SUCNR1 (NBP1-00861, Novus Biologicals), HIF-1α (D1S7W) (36169, Cell Signaling Technology), Cytokeratin 14 (ab7800, Abcam), CD8 (10980-MM38, SinoBiological), Cytokeratin 14 (60320-1-Ig, Proteintech), CXCL9 (ab320827, Abcam), or CXCL10 (10937-1-AP, Proteintech) overnight at 4 °C. After washing with PBS with Tween-20 (PBST), sections were incubated with corresponding secondary antibodies (1:1000) (Goat anti-Rabbit IgG Cy3®, ab6939, Abcam; Goat anti-Mouse IgG Alexa Fluor® 488, ab150113, Abcam) at room temperature for 1 h away from light. After washing with PBST, the nuclei were counterstained with DAPI (1:1000, C1006, Beyotime Biotechnology) at room temperature for 10 min in the dark. Images were acquired by laser confocal microscopy (LSM510, Carl Zeiss AB).

Enzyme-linked immunosorbent assay (ELISA). Cell culture supernatant was collected and centrifuged to remove particulates. QuantiCyto® Human MIG/CXCL9

ELISA kit (EHC114, NeoBioscience), QuantiCyto® Human IP-10/CXCL10 ELISA kit (EHC157, NeoBioscience), and QuantiCyto® Human CXCL16 ELISA kit (EHC168, NeoBioscience) were used to measure supernatant CXCL9, CXCL10, and CXCL16 concentration according to the corresponding manufacturer's instructions. In brief, 100 μL diluted standards and samples were added to the wells and incubated for 90 min. Next, the supernatant was discarded, and the wells were washed with 300 μL wash buffer. Then, 100 μL biotinylated antibody working solution was added into each well and incubated for 60 min. After washing, 100 μL enzyme conjugate working solution was added to each well and incubated for 30 min. 100 μL substrate solution was added to each well post-washing, followed by a 15 min incubation. Subsequently, 100 μL stop solution was added to each well and the absorbance was determined at 450 nm by Model 680 Microplate Reader (Bio-Rad). Concentrations of CXCL9, CXCL10, and CXCL16 in samples were calculated from a standard curve. All incubation processes were at 37 °C protected from light.

Transwell migration assay. A density of 1×10^5 $CD8^+$ T cells in 100 μL medium was plated in the upper chambers of a transwell chemotaxis 24-well chamber with a 5.0-μm polycarbonate membrane (3421, Corning). 300 μL culture supernatants of NHKs with or without recombinant human CXCL9 (392-MG, R&D Systems) and CXCL10 (266-IP, R&D Systems) were added to the lower chambers. After incubating

the cells at 37 °C for 3 h. The number of migrated CD8$^+$ T cells in the lower chamber was detected by flow cytometry.

Chromatin immunoprecipitation (ChIP) assay. ChIP assay was performed using the SimpleChIP Plus Sonication Chromatin IP Kit (56383, Cell Signaling Technology) according to the manufacturer's instructions. Briefly, cells were fixed with 1 % formaldehyde, the chromatin was fragmented by ultrasound to obtain chromatin fragments of 200-1,000 bp. Chromatin immunoprecipitation was performed using HIF-1α (D1S7W) (36169, Cell Signaling Technology) and ChIP-Grade Protein G Magnetic Beads. After protein-DNA decross-linking, DNA was purified using a DNA purification centrifuge column. The purified DNA and input DNA were analyzed by qRT-PCR. % Input was calculated as follows: 2 % $\times$ 2^(Ct^{Input}-Ct^{Sample}).

A 2,000 bp sequence upstream of the CXCL9 gene was considered to be the promoter region of the CXCL9 gene as shown here:

TAGCTAGTCAACCAAAATAAAGATGACGTTTCTTAACTTCCCCTACAACTA
GGTGTAGCCATGTGATTAAGGCCTGCCTCCATGGGAGGTGATAGAAAATG
ATGGGTGCAACTTCTAGGATGTCTAAAAAGGAGGGACATGCCCTTTTTCT
GCCTTCTCATCATCCTATACCTGGAAATTGGACATGGTGGCAAGCCACCTT
TGGCCATGTGCATGAGGGCAGCAATCTAGAGATGGTGACAACACAAGAA
AGGAGAAGCCTGGAAGACCTCATGGGAAAGAGCCACTATCCAAGTGCTA
ATCCACCTACATCTCTCAGGGAGAAATAAGCTTCTATCTTAGCCACTGTTT

TCTGTTGTTGTTTTTTTTTTTTTTATTTTTCCATAAGTTATTGGGGTACAGGT

GGTATTTGGTTACACGAATAAGTTCTTTAGTGGTGATTTGTGAGATTTCGG

TTCACCCATCACCCAAGCAGTATACACTGCACCCTATTTGTAGTCTTTTAT

CCCTCACCCCGCTCCCACTCTTCTCCACAAGTCCCCAAACTCCATTGTATC

ATTCTTATGCCTTTGTGTCCTCATAGCTTAGCTCCCACATACCAGTGAGAA

CAGATGATGATTGGTTTTCCATTCCTGAGTTACTTCACTTAGAATAATAGT

CTCCAATCTCATCCAGGTCACTGCTAATGCCGTTAATTCATTCCTTTTTGTG

ACTGAGTAGTATTCGATTGTGTATATATAAACCACAGTTTCTTTATTCACT

CATTGATTGATGGGCATTTGGGTTGGTTCCATGATTTTGCAATTGTGAATT

GTGCTGCTATAAACATGCATGTGCAAGTACATTTTTCAAATAATGACTTCT

TTTCCTCTGGGTAGATACCCAGTAGTGGGATTGCTGGATAAAATGGTAATT

CTGCTTTTTAGTTCTTTAAGGAATCTCCACACTGTTTTCCATAGCGGCTGTG

CTAGTTTACATTCCCACCAGCAGTGTAGAAGTGTTCCCTGATCACCAAGTC

CACACCAACATCTGCTGTTTTTTGATTTTTTGATTATGGCCATTCTTGCAGG

TGTAAGGTGGTATCACACCGTGGTTTTGATTTGCATTTCCCTGATCATTAG

TGATGAGCATTTTTTCATATGTTTGTTGGCTATTCGTGTATCTTCTTTTGAG

AATTGTATATTGATGTCCTTAGCCCACTTTTTAATGGGATTGTTTGTTTTTT

ACTTAATGATTTGTTTGAATTCATTCTAGATTCTGGATATTAGTCCTTTGTC

AGGTATATAGATTGTGAAGATTTTCTCCCACTCTGTGGGTTGTCTGTTTAC

TCTGCTGACTGTTCCTTTTGCTGTGCAAAAGCTCTTTAATTAGGTCCCAGC

TATTTATCTTCATTTTTATTGCATTTCTTAGCCACTGTTAATTTGGGTCTCTT

ACATGCAACTGACCCCTTATTCTAACCAATTTATTAGTTTAAAAAATAATA

CTGTAGCAGGAATGATACAGAATGAGCTTACCACTAGTAGAAAATGACCT
GGTCCCAGAGGAAATTAAGGTGCTTTAAGTTGTTAAGTGGGCAATATAAG
AAAATATCAATGTAGCCATCTATTATACAGAAGAATATGCCATAAATAGT
AAGGGAATTTGAAAAGTATATTATTTATCTGGACTTTAATATTTCCCATCT
GGAAACCACATCTCCAACCTTTGTTGTATAACATTAGTATATTCATTATAT
AACCTATTCTTCACACAGGTGAGCAACTGTGAATGTGCCAAAGGCTATCA
GTGTTGCTAGTTTCACATCCCTTACTATAAACCACCCATTTATGTGAAATG
GAAATAAAAACATGCAGAAATTCCCTTGGATCTGAGACTAGGGTTTCCCC
CAGCACAATCAAGTTTGTGGTCAATTTAGTTAGCTCAGCTAAAGATACTA
GCCAATCAGAGTTTAGGAGAGAAATGAATATCCTAAATAAATATGATCCC
CAAAATGTGTTCTCT.

A 2,000 bp sequence upstream of the CXCL10 gene was considered to be the promoter region of the CXCL10 gene as shown here:

CACAGTTAATGTAATACAATGTTTAGTAAAAATCAAATATTTTATCCCTAA
TAACAGATTTTTAGTCCATTTATCAAGTATCTCCTTTTTACATTGTCTTCTA
TATAGAGACATTTTAGCTGACAACTTAGATACCAACTAAAATAAAACTGT
CACCATCTCTCATTTTGATTGTTTAATGTTGAACTTTTCTAGGCCTCTTCCA
TTCATGATGTCAGACATTTCTCTGAAATTTGTTGGCATTCTATTCACAGTA
AAATGTTTGTTCTGCACAAATAGGTATGGAAGGATCCCTCCATTGTCACTA
TTTCTCATAAGACTTTCCCTCATCAGAACGAACGCCTCTTTGCTAGGTGAA
AGTCTTTTGGGTTCTGTCAGTCTCTACCTCACCTCCCTCTCTTTTGCCTTTC

CGGTTTCCCACAGCTAATTCCACTGACTTATTCACTTGAAAGCTTAGCTCT

ATTCCAGTTCCTAGAACAGATTAACATGGGATTCAGCTCTCTAGCAAATTC

CATTTACCCCAAGCAACTCACTCTCTGCTTTAATGATGGAGTTCCTACATT

TGAGGATCCAAGTTTTATGTGCACCCAGTTTGCAGATACAGGCTTTATTCT

TGGCCCCTGATACCAGGTTCTGAGACAGTATCTGCCTAGTACAGTAGCCT

GATCCTAGGATGGAAGCTTTTCCATTCCCTGACCAATCCCTGCATTGTTGC

AGCCCAGCTGACTATGAAAAGAACTCCCATATATTTTCCTAGGTTCTAAAT

ATCACTTGCCTCAGATCTCTCATTGCAGGGTGGTGCCTGCCTTTGAAACAG

TATTTCATCTTGGATTCCCTGTTACTAATCATTTGCAAAGAACACAACCAA

GGACCACTATATCCATGGATACTGCTAAATTTCCACTCTCAGGTTCAACAT

GTGTTCCATTATATGAGTATCATGTGCTTTCTACTGCTAGCTACTATGAAT

AATCAGTCAAGTACTATTGAATGCTGTGAAATTAAGTTTTGCCACGATTCA

TCATCCAGTTAAAATTGTGCCAATTTCAGTTATCACTGTTACTAGCTCAAT

GGCCAACACTTTTTTTTTCTTTTTCTTTGCATTTAACAGTGTCTTGGAGCTG

AACCCCATCGTAAATCAACCTGTTTCCCTTCTGTCTCAAGCTACTTTCCCA

AGGTCTGTCTCTATGCGTGCCCCCCCGCTTTTAAATTCATTTCCTCAAAAA

GCACCCTTTGTTTTTTACAATAACCCTAGGATAGCTATGAATCCTTTTCTC

ACACCATTCTAGATACCATCTTATGATGTTTTCATTCAGGGACTGCTATAA

GACGTGAAACTTGTTTTAACACACCACAAATCAGATACCCAAATGAGCAA

TGTTTTCCCTCAAAATAGTTATGTTGGAGGCTATTTACTTAATCTAATGGT

GTTATGAATGATCAAAGCATTTATTGAATTATTTAAAACTTGCCAGTTCCA

GATCTTTGACCCTGTCAAAGCAGGCCAGTCCTATTACTTTATAGTATTTGA

TCAAGGAGGACTGTCCAGGTAAATCACTGTTCTAATAATCAGGCACAACT
TGCTGTTACCAAAAAATTAGGTTTACCTATAAAGGATGAAAAATTTCTATT
ACTGGGGATATTTTGAAAAATAACCTCTGAAAGTAACTGTAAAAGTTTAA
GTGTTGAGAAAAAGCATTATAGTTAGAATGGATTGCAACCTTTGTTTTTTT
CTATATGCAATGAAGTTCTTTTTTTCAAGAAACAGTTCATGTTTTGGAAAG
TGAAACCTAATTCACTATTACCAAAAAAAGAGGAGCAGAGGGAAATTCC
GTAACTTGGAGGCTACAATAAATAATACCTTCGAGTCTGCAACATGGGAC
TTCCCCAGGAACAGCCAGCAGGTTTTGCTAAGTCAACTGTAATGCCCTTAT
CCAATCAGAATTAGGGAGGGAAAATGGCTTTGCAGATAAATATGGCACAC
TAGCCCCACGTTTTCT.

The primer sequences used were as follows:

Primer 1 of *CXCL9* (forward 5'-TTCTTTAGTGGTGATTTGTGAGAT-3', reverse 5'-GAATGAATTAACGGCATTAGCA-3'), Primer 2 of *CXCL9* (forward 5'-ATGGGCATTTGGGTTGGTTCCATG-3', reverse 5'-TCTTCACAATCTATATACCTGA-3'), Primer 3 of *CXCL9* (forward 5'-AAATAGTAAGGGAATTTGAAAAG-3', reverse 5'-CTAAATTGACCACAAACTTGA-3'), Primer 1 of *CXCL10* (forward 5'-GCCACGATTCATCATCCAGT-3', reverse 5'-TAGCAGTCCCTGAATGAAAACA-3'), Primer 2 of *CXCL10* (forward 5'-ATTCATTTCCTCAAAAGCACCC-3', reverse 5'-GTAACAGCAAGTTGTGCCTGAT-3'), and Primer 3 of *CXCL10* (forward 5'-

TTCGAGTCTGCAACATGGGA-3’, reverse 5’-GAGTCAGAAAGATAAGGCAGCA-3’).

Co-immunoprecipitation (Co-IP) assay. Cells were treated with 10 μM MG-132 (HY-13259, MedChemExpress) before harvesting. After washing with PBS three times, cells were lysed in RIPA lysis buffer (P0013C, Beyotime Biotechnology) containing 1 % PMSF (ST506, Beyotime Biotechnology) at 4 °C for 20 min. The supernatant was then collected by centrifugation at 12,000 rpm for 15 min at 4 °C to extract total cellular protein. With a portion of the total cell lysate saved as input sample, the remaining lysate was precleared with Protein A/G PLUS-agarose (sc-2003, Santa Cruz Biotechnology) before incubation with anti-6*His, His-Tag antibody (66005-1-lg, Proteintech) or normal mouse IgG (A7028, Beyotime Biotechnology) at 4 °C overnight. Protein-bound beads were then collected by centrifugation and washed four times with ice-cold RIPA buffer. Immunoprecipitates were resuspended with 1×loading buffer and boiled for western blotting assay with primary antibodies against His (66005-1-lg, Proteintech), K48-Ub (ab140601, Abcam), and PHD2 (NB100-137, Novus biological) and corresponding secondary antibodies (Mouse Anti-rabbit IgG (Conformation Specific) (L27A9) Antibody, HRP Conjugate, 5127, Cell Signaling Technology; Rabbit Anti-Mouse IgG (Light Chain Specific) (D3V2A) Antibody, HRP Conjugate, 58802, Cell Signaling Technology).

Targeted liquid chromatography-mass spectrometry (LC-MS) metabolomics analysis. Add 1 mL of ethyl acetate to 100 μL serum, ultrasonically-assisted extraction in an ice-water bath for 30 min. After centrifugation (12,000 rpm, 5 min), the supernatant was neutralized with concentrated hydrochloric acid and extracted two times with diethyl ether. 35 μL of extraction solution was dispensed into a 96-well plate, and then 100 μL of methanol (containing internal standard solution) or external standard solution was added and vortexed for 10 min. The eluate was collected and concentrated by nitrogen blowing. Subsequently, a mixture of 70 % acetyl chloride and 30 % n-butanol (v/v) was added to the dried samples. After derivation, centrifugation, and filtration, the samples were determined by liquid chromatography coupled to a tandem mass spectrometer. The concentrations of metabolites in the serum were calculated using the external standard method. Data were calculated and analyzed using ChemView software (AB SCIEX). For data quality analysis, distance to model X (DModX) was used to inspect the outliers using the SIMCA software. The threshold for a moderate outlier is considered when the sample DModX value is twice the DCrit at p-value = 0.05. Orthogonal partial least squares-discriminant analysis (OPLS-DA) and variable importance in projection plot (VIP plot) (VIP > 1.0 was used as subsequent screening criteria) were performed using the MetaboAnalyst 5.0 (https://www.metaboanalyst.ca/). The Mann-Whitney U test and Spearman rank correlation test were performed in SPSS version 22.0.

Single-cell RNA-seq analysis. Publicly available single-cell RNA-seq data were downloaded from the Genome Sequence Archive (GSA) (accession numbers PRJCA006797 and GSE231794). The gene expression data were mapped using CellRanger (v.2.1.1 and v.2.0.2) to an Ensembl 84-based GRCh38 reference (10x Genomics–distributed v.1.2.0). The Python package emptydrops (v.0.0.5) was used to detect cells in each sample. Potential doublets were flagged using Scrublet (v.0.2.1). Low-quality cells were filtered out first by using a median + (X × MAD) score (where MAD is the median absolute deviation) of the median score for the mitochondrial UMI fraction (5 × MAD), maximum number of UMIs (8 × MAD), followed by strict cut-off values (minimum number of genes = 200, maximum number of UMIs = 50,000, maximum mitochondrial UMI fraction = 0.20). Louvain clusters were manually annotated using marker genes identified through the literature search, and their expression of distinctive DEGs specific to each cluster. Differential analysis of SUCNR1 and other genes was performed using a Wilcoxon signed-rank test.

Statistics. Data analysis was performed using GraphPad Prism version 9.0 software. Comparison of categorical data was performed by using the chi-square test. Shapiro-Wilk test and Levene's test were used to assess the normality of distribution and homogeneity of variance. Dual comparisons were analyzed with the Student's *t*-test, Mann-Whitney *U* test, or Wilcoxon signed-rank test. Groups of three or more were analyzed by using one-way analysis of variance (ANOVA) with Tukey posttests

or Kruskal-Wallis *H* test with Bonferroni posttests. Data are shown as median ± interquartile range (IQR) or mean ± standard error of the mean (SEM). Spearman rank correlation analysis was employed for data with non-parametric distribution for the correlation analysis. *p*-values less than 0.05 were considered statistically significant. Each experiment was independently repeated at least three times.
