# Supplementary material for: Hyperglycemia aggravates vitiligo through succinate/SUCNR1-mediated T cell activation
Source: J Clin Invest. 2026 Jun 15;136(12):e200316. doi: 10.1172/JCI200316 (PMC13262724; doi:10.1172/JCI200316)
Supplement: Unedited blot and gel images [file jci-136-200316-s186.pdf]

Full unedited blot images for

Hyperglycemia aggravates vitiligo through succinate/SUCNR1-mediated T cell activation

Pan Kang[1], Yuqian Chang[1], Tingting Wang[1], Xiuli Yi[1], Yinghan Wang[1], Pengran Du[1], Jiaxi Chen[1], Baizhang Li[1], Shuli Li[1], Zhongjun Shao[2], Jianru Chen[1,3], Chunying Li[1]

Correspondence: Chunying Li (lichying@fmmu.edu.cn), Jianru Chen (chenjr0516@fmmu.edu.cn)

Full unedited blot for Figure 4B

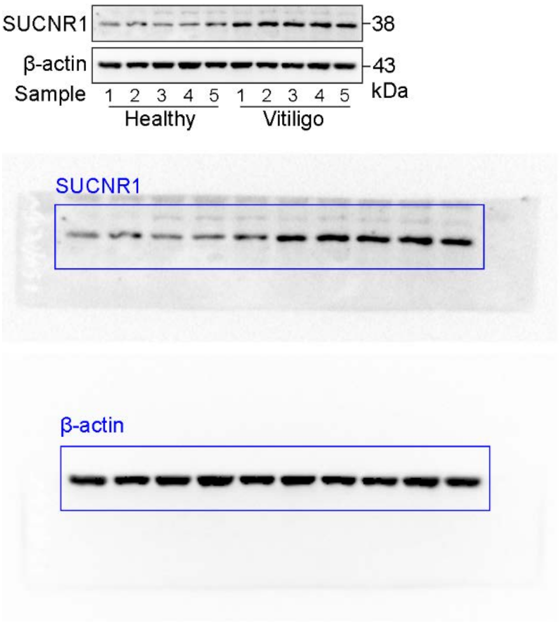

Full unedited blot for Figure 4F

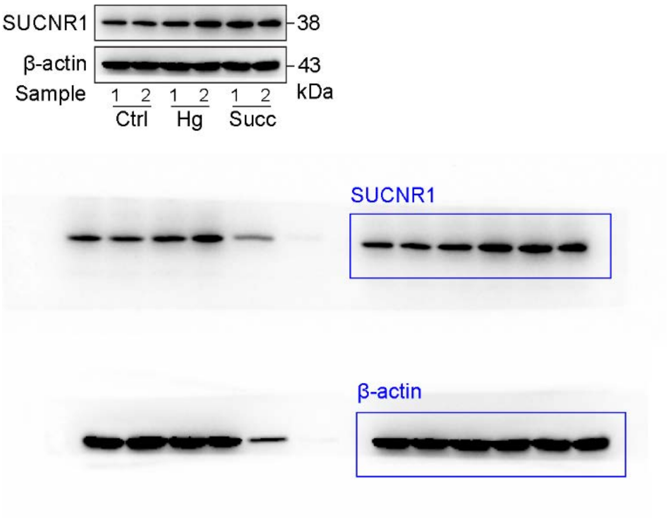

Full unedited blot for Figure 9B

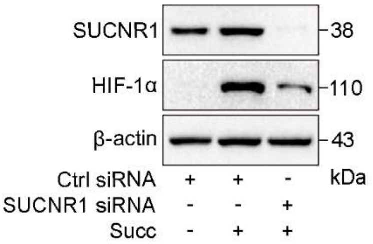

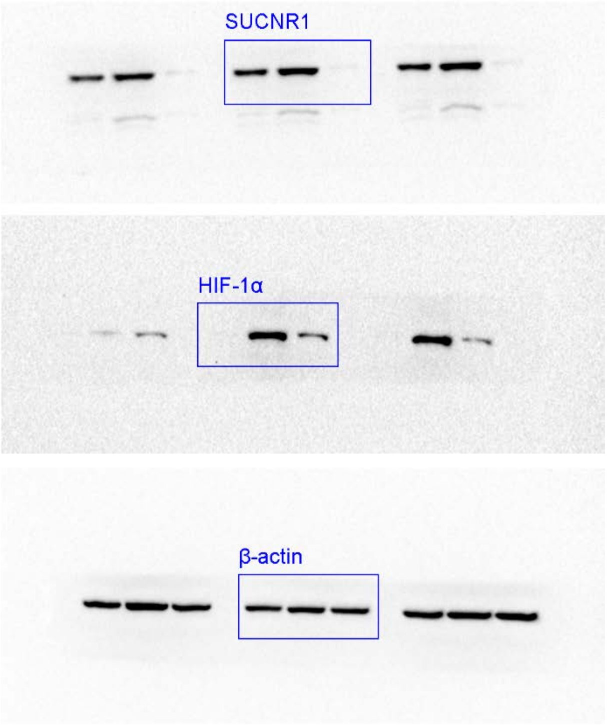

Full unedited blot for Figure 9E

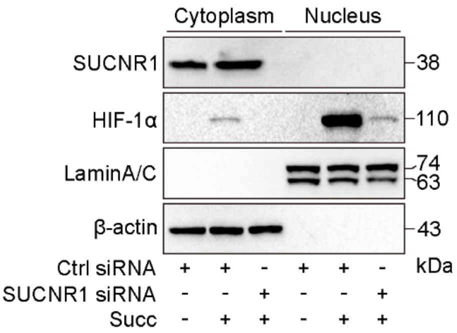

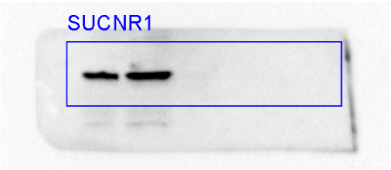

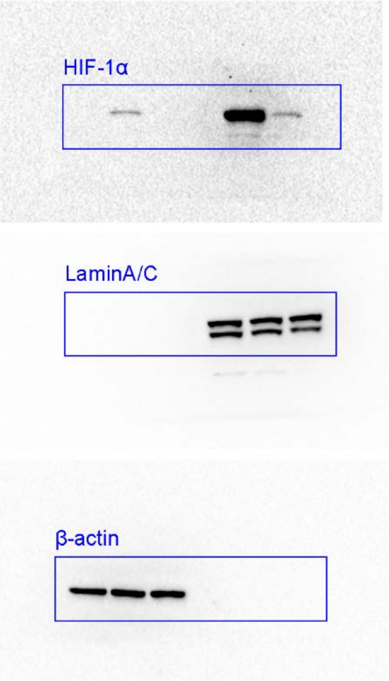

Full unedited blot for Figure 10B

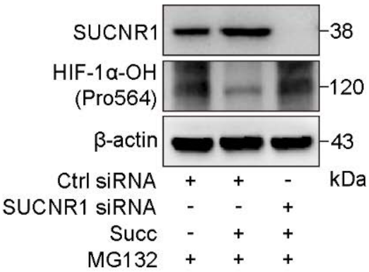

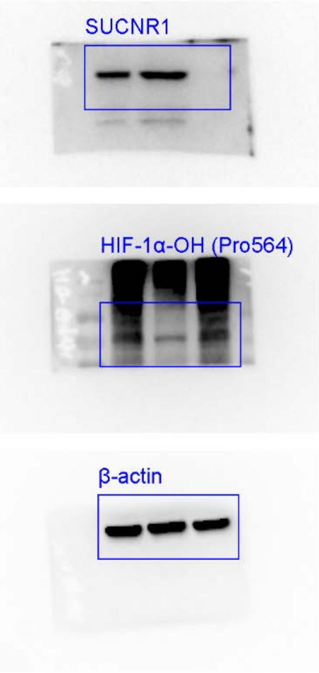

Full unedited blot for Figure 10C

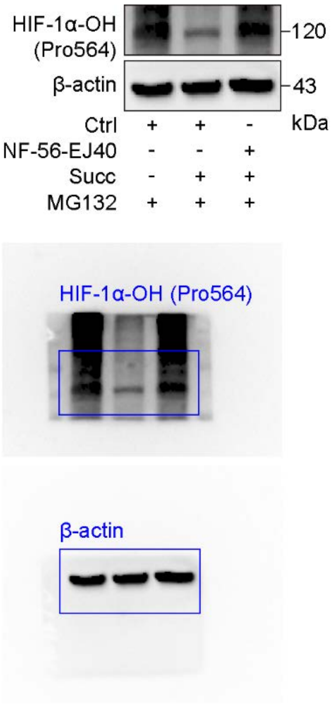

Full unedited blot for Figure 10D

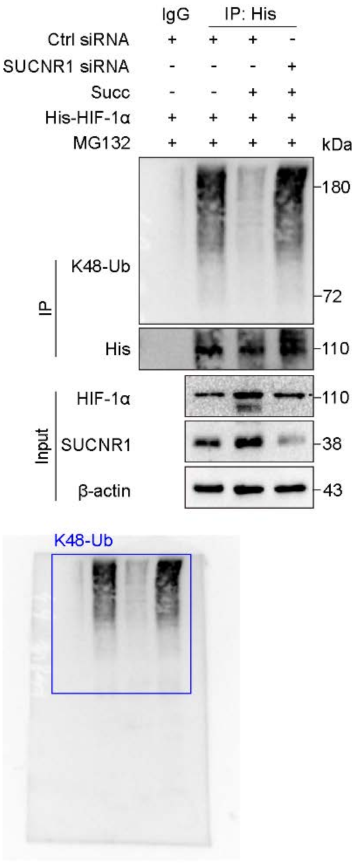

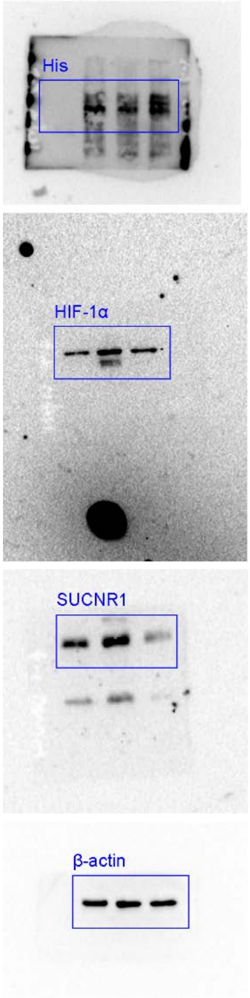

Full unedited blot for Figure 10E

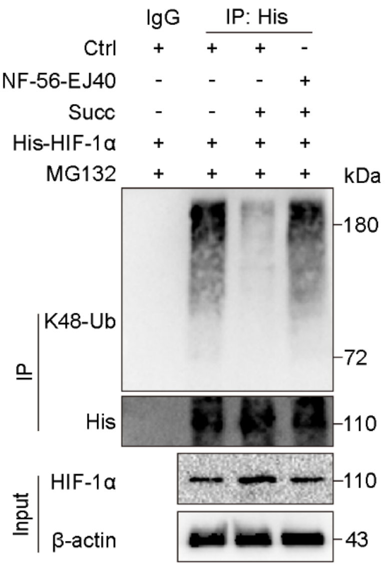

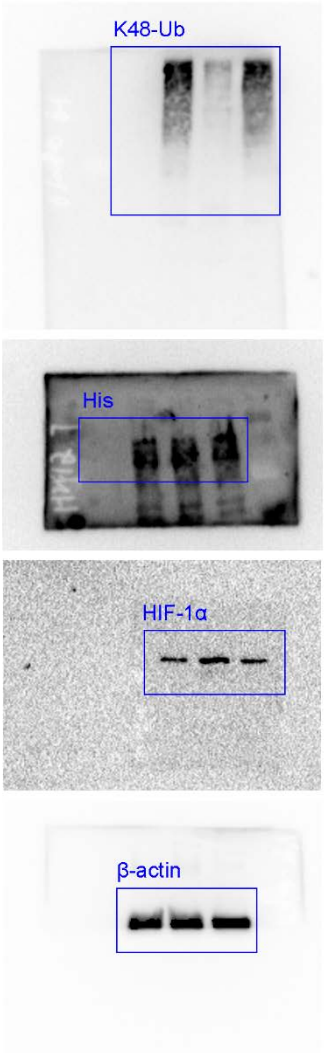

Full unedited blot for Figure 10F

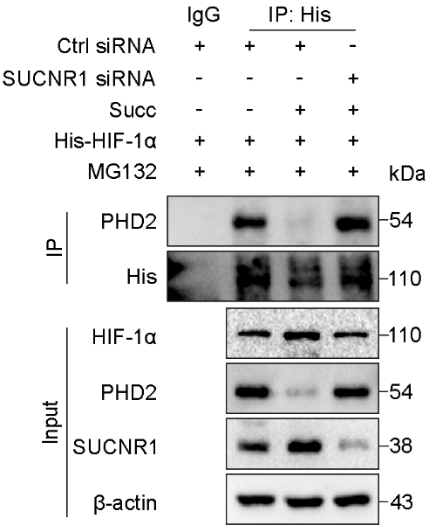

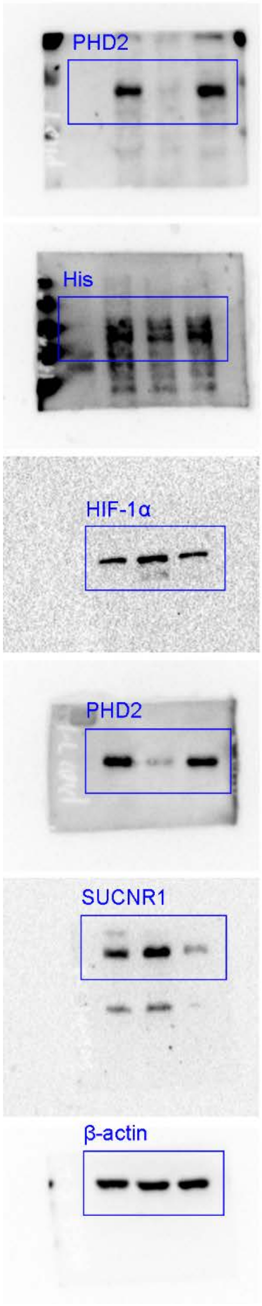

Full unedited blot for Figure 10G

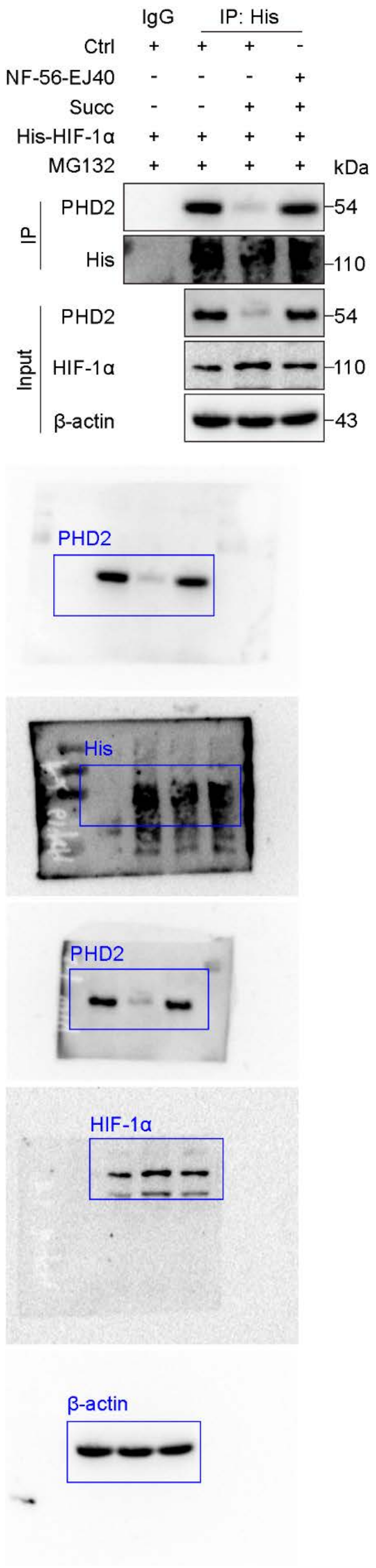

Full unedited blot for Figure S11B

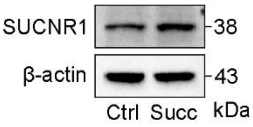

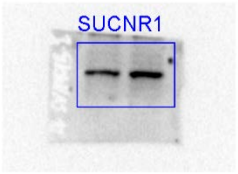

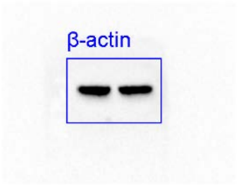

Full unedited blot for Figure S12A

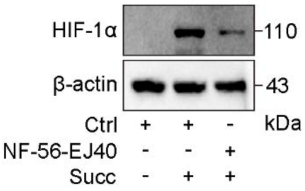

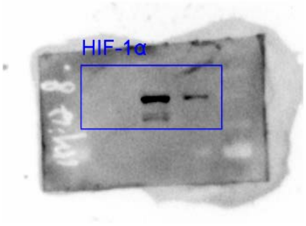

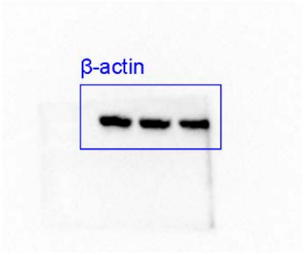

Full unedited blot for Figure S12B

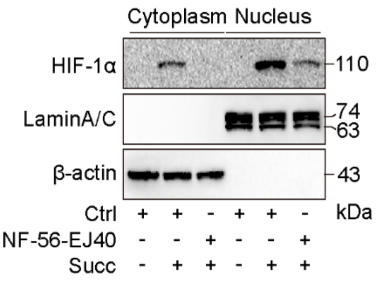

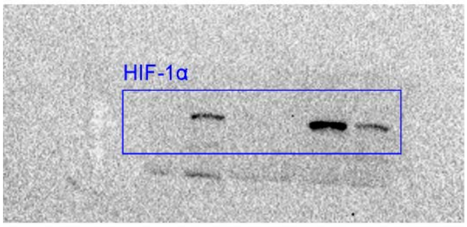
HIF-1α

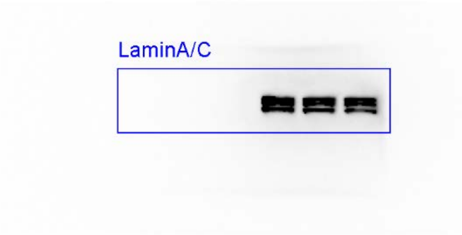
LaminA/C

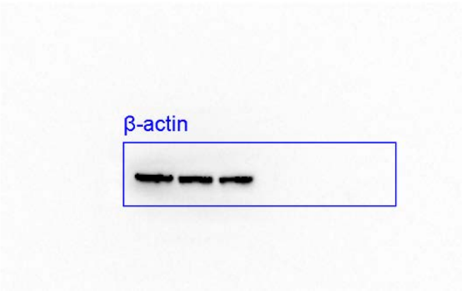
β-actin
